# A new Early Oligocene toothed ‘baleen’ whale (Mysticeti: Aetiocetidae) from western North America: one of the oldest and the smallest

**DOI:** 10.1098/rsos.150476

**Published:** 2015-12-02

**Authors:** Felix G. Marx, Cheng-Hsiu Tsai, R. Ewan Fordyce

**Affiliations:** 1 Department of Geology and Palaeontology, National Museum of Nature and Science, Tsukuba, Japan; 2 Department of Geology, University of Otago, Dunedin, New Zealand; 3 Departments of Paleobiology and Vertebrate Zoology, National Museum of Natural History, Washington DC, USA

**Keywords:** Mysticeti, baleen whale, Aetiocetidae, suction feeding, filter feeding, baleen

## Abstract

Archaic toothed mysticetes represent the evolutionary transition from raptorial to bulk filter feeding in baleen whales. Aetiocetids, in particular, preserve an intermediate morphological stage in which teeth functioned alongside a precursor of baleen, the hallmark of all modern mysticetes. To date, however, aetiocetids are almost exclusively Late Oligocene and coeval with both other toothed mysticetes and fully fledged filter feeders. By contrast, reports of cetaceans from the Early Oligocene remain rare, leaving the origins of aetiocetids, and thus of baleen, largely in the dark. Here, we report a new aetiocetid, *Fucaia buelli*, from the earliest Oligocene (*ca* 33–31 Ma) of western North America. The new material narrows the temporal gap between aetiocetids and the oldest known mysticete, *Llanocetus* (*ca* 34 Ma). The specimen preserves abundant morphological detail relating to the phylogenetically informative ear bones (otherwise poorly documented in this family), the hyoid apparatus and much of the (heterodont) dentition. *Fucaia* comprises some of the smallest known mysticetes, comparable in size with the smallest odontocetes. Based on their phylogenetic relationships and dental and mandibular morphology, including tooth wear patterns, we propose that aetiocetids were suction-assisted raptorial feeders and interpret this strategy as a crucial, intermediary step, enabling the transition from raptorial to filter feeding. Following this line of argument, a combination of raptorial and suction feeding would have been ancestral to all toothed mysticetes, and possibly even baleen whales as a whole.

## 1. Introduction

Aetiocetids are a clade of archaic toothed mysticetes known exclusively from the Oligocene [1], although recent, as yet unpublished, reports may hint at a possible survival until the late Early Miocene [2]. Originally regarded as archaeocetes [3], aetiocetids were subsequently recognized as basal mysticetes [4] and recently gained prominence as potential morphological intermediates between modern baleen-bearing mysticetes (Chaeomysticeti) and their toothed ancestors [5,6]. At present, Aetiocetidae comprise eight species in four genera—*Aetiocetus*, *Ashorocetus*, *Chonecetus* and *Morawanocetus*—from both sides of the North Pacific basin [1]. *Willungacetus aldingensis* from the Early Oligocene of Australia has also been referred to this family [7], but the material is too poorly preserved to allow confident identification [8]. In stark contrast, other toothed mysticetes, including mammalodontids and llanocetids, are only known from the Southern Hemisphere [8–10], with the possible exception of a potential, fragmentary mammalodontid from Malta [11].

Apart from *Willungacetus* and a referred Early Oligocene specimen of *Aetiocetus cotylalveus* (USNM 256593 [6]), all reported aetiocetids date from the Late Oligocene. Considering their intermediate morphology, this temporal distribution is rather striking, given that (i) the earliest toothed mysticete, *Llanocetus denticrenatus*, is known from the latest Eocene (*ca* 34 Ma [12]); and (ii) even chaeomysticetes are attested from the late Early Oligocene onwards [13]. Nevertheless, it is likely that the apparent lack of Early Oligocene aetiocetids reflects a patchy fossil record, as shown by the mere handful of cetacean specimens from this period, rather than a genuine biological phenomenon. Here, we reduce this temporal gap by describing a new species and genus from the Early Oligocene Makah Formation of the Olympic Peninsula, WA, USA. The new material represents the oldest reported aetiocetid, and, crucially, preserves details of the otherwise poorly known dental, ear bone, hyoid and vertebral morphology characterizing this family. In particular, our specimen stands out from other reported aetiocetids in including an almost entirely preserved, extracted periotic, some well-preserved tooth crowns showing details of wear, and all of the cervical vertebrae. The new species represents one of the smallest mysticetes yet described and thus highlights the rather humble origins of the giants that plough the modern oceans.

## 2. Material and methods

Most of the preparation of the new material was done mechanically and carried out at the Burke Museum of Natural History and Culture, University of Washington, USA. Additional preparation, including the extraction of the right periotic and details of the tooth crowns, was carried out at the Geology Museum of the University of Otago (Dunedin, New Zealand) using dilute acetic acid and pneumatic air scribes under a Zeiss binocular microscope. Morphological nomenclature and tympanoperiotic orientation follow Mead & Fordyce [14], unless indicated. We repeated the dated total evidence phylogenetic analysis of Marx & Fordyce [13], fig. 2, which already included the material described here under the label UWBM 84024. We retained all the taxa, scorings and analysis settings of the previous analysis, but made four alterations to the data matrix.

First, we modified character 33 (fusion of the posterior premolar and molar roots) so that it now includes three ordered states: (0) roots separate along their entire length; (1) roots fused proximally, but separate distally; (2) roots fused or closely apposed along their entire length. This change was made to reflect the disparate morphology of *Morawanocetus* and mammalodontids (partially fused roots) and *Aetiocetus* and *Fucaia* (completely fused or apposed roots) in this regard. Second, we changed the coding of character 29 (enamel ornament on premolars) for *Morawanocetus*, from state 0, ‘vertical enamel ridges present on lingual surface only’, to state 1, ‘enamel ridges present on both lingual and labial surfaces’. Finally, we changed the codings of characters 191 (*in situ* orientation of main axes of tympanic bullae in ventral view) and 243 (parapophysis on seventh cervical vertebra) for *Fucaia*, from state 0, ‘diverging posteriorly’ to ‘?’, and from state 0, ‘present’ to state 1, ‘absent’, respectively. The latter three changes were made to correct previous coding errors (see discussion of phylogeny below).

Our new morphological codings and the full matrix are available from MorphoBank, project 2238 (full matrix stored in the ‘Documents’ section). The analysis of the amended data matrix was run in MrBayes v. 3.2.6 [15], on the Cyberinfrastructure for Phylogenetic Research (CIPRES) Science Gateway [16]. In addition, we analysed the morphological partition of our data using the traditional search option of TNT (v.1.1) [17,18], based on 5000 random stepwise-addition replicates and tree bisection reconnection branch swapping, saving 10 trees per replicate. Parsimony analyses were run assuming both equal and implied weighting (*k*=3), with branch support estimated through symmetric resampling (2000 replicates), recorded as GC values [19].

### 2.1 Institutional abbreviations

AMP, Ashoro Museum of Paleontology, Ashoro, Hokkaido Japan; ChM, Charleston Museum, Charleston, South Carolina, USA; CMN, Canadian Museum of Nature, Ottawa, Canada; LACM, Natural History Museum of Los Angeles County, Los Angeles, CA, USA; NMV, Museum Victoria, Melbourne, Australia; OCPC, John D. Cooper Archaeological and Paleontological Center, Santa Ana, CA, USA; OU, University of Otago Geology Museum, Dunedin, New Zealand; USNM, National Museum of Natural History, Smithsonian Institution, Washington, DC, USA; UWBM, Burke Museum of Natural History and Culture, University of Washington, Seattle, WA, USA.

## 3. Systematic palaeontology

Cetacea Brisson, 1762

Mysticeti Gray, 1864

Aetiocetidae Emlong, 1966

*Fucaia*, gen. nov.

*LSID.* urn:lsid:zoobank.org:act:FDBB95FF-5D4F-4DD2-95F7-C834D07442FA

*Type species*. *Fucaia buelli*, sp. nov.

*Etymology*. After the Strait of Juan de Fuca, the area surrounding which yielded both *F. buelli* and its sister species *F. goedertorum*.

*Diagnosis*. Small-sized mysticetes (approx. 2 m in length) differing from all chaeomysticetes in bearing teeth. Differ from *Llanocetus*, *Morawanocetus* and mammalodontids in lacking labial enamel ornament; from *Llanocetus* in their much smaller size and in having a proportionally larger, more anteriorly directed orbit, a shorter nasal and a straight (rather than concave) rostral margin; from mammalodontids in having a transversely wider intertemporal region, a mandible with a centrally constricted body (in lateral view) and a comparatively elongate rostrum; from *Janjucetus* in having an unfused mandibular symphysis; from *Aetiocetus* and *Morawanocetus* in having a distinctly V-shaped fronto-parietal suture in dorsal view, an anteroposteriorly longer intertemporal region, and a relatively short and stocky posterior process of the tympanic bulla; from *Aetiocetus* in having a clearly heterodont dentition, a narrower rostrum and a dorsally flattened braincase (in lateral view); from *Morawanocetus* in having a much more robust postorbital process of the frontal and more gracile cheek teeth; from *Chonecetus* in having an anteriorly pointed supraoccipital shield bounded by anterolaterally flared nuchal crests, an external occipital crest, a flattened skull table defined by parasagittal crests and in lacking parasagittal clefts; and from *Ashorocetus* in having a less steeply inclined supraoccipital and a short and stocky compound posterior process of the tympanoperiotic. Finally, *Fucaia* differs from the enigmatic *Willungacetus* in having a transversely narrower supraoccipital bearing a better-developed external occipital crest, a more posteriorly projected nuchal crest and a flattened skull table defined by parasagittal crests.

*Remarks*. Comparisons with *Chonecetus sookensis*, *Ashorocetus* and *Willungacetus* are hampered by the poor state of preservation of the available material. This especially applies to *Ashorocetus*, which is currently only known from the fragmentary posterior portion of a braincase [1]. Based on the lack of diagnostic characters, Fitzgerald [8] reclassified *Willungacetus*, previous tentatively referred to Aetiocetidae [7], as Mysticeti incertae sedis and proposed that *Ashorocetus* may represent a *nomen dubium*. We concur with this assessment, pending the discovery of better-preserved material that could help to clarify relationships.

*Included taxa*. *Fucaia buelli*, sp. nov.; *Fucaia goedertorum*, comb. nov.

*Fucaia buelli*, sp. nov.

(figures 2–17)

*LSID*. urn:lsid:zoobank.org:act:576499AC-3F38-43EC-9D69-542ED65E35B3

*Holotype*. UWBM 84024, partial skeleton comprising the cranium including both periotics and the right tympanic bulla, a part of the right mandible, 17 isolated teeth, most of the hyoid apparatus, 20 vertebrae, part of the left scapula, a heavily eroded radius, and several non-diagnostic, partially prepared fragments.

**Figure 1.**
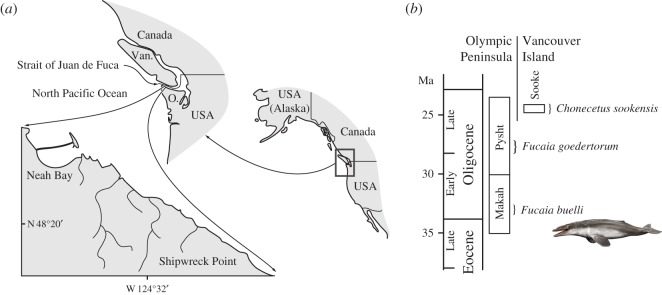
Type locality and horizon of *Fucaia buelli*. (*a*) Locality map, (*b*) age and provenance of *Fucaia* and *Chonecetus*. Details as to the exact location and horizon are available directly from UWBM. O., Olympic Peninsula; Van., Vancouver Island.

*Locality and horizon*. UWBM Locality C716, between Shipwreck Point and Neah Bay, Clallam County, Olympic Peninsula, WA, USA (figure 1). The specimen was collected by J.L. Goedert and B.R. Crowley as a concretion less than 1 m in length, derived from siltstone forming part of the Makah Formation (either the Jansen Creek Member or the horizon immediately below). Details as to the exact location and horizon are available directly from UWBM.

The upper portion of the Makah Formation has been correlated with the local Zemorrian Foraminiferal Stage based on the presence of *Uvigerina gallowayi*, among others [20]. By contrast, the lower portion of the formation appears to fall into the Refugian, as indicated by the occurrence of *Sigmomorphina* cf. *S. schencki*, *Cassidulina* cf. *C. kernensis* and *Ceratobulimina washburnei* [20], although at least *Ce. washburnei* may range above the Refugian/Zemorrian boundary [21]. The Zemorrian spans almost the entire Oligocene and, at least in the area surrounding the Strait of Juan de Fuca, cannot easily be distinguished into early and late substages [22]. Nevertheless, magnetostratigraphic data place a large portion of the Makah Formation, including some of the Refugian horizons and the Jansen Creek Member, into a single reverse polarity zone. Given the presence of Refugian foramina in its lower portion, and assuming that there are no major gaps, the most likely correlation of this magnetozone is with Chron C12r, implying an age of approximately 33.2–31.0 Ma, or Early Oligocene [23,24].

*Etymology*. Named after Carl Buell, in honour of his artistic achievements in illustrating extant and fossil cetaceans.

*Diagnosis*. Small-sized mysticete (approx. 2 m in length) corresponding in all preserved features with the diagnosis of *Fucaia*. Differs from *F. goedertorum* in having a more elevated posterior portion of the nuchal crest (and thus a more concave supraoccipital shield), an ascending process of the premaxilla that is narrower than the ascending process of the maxilla, an ascending process of the maxilla that extends as far posteriorly as the nasal, a clearly defined (as opposed to interdigitating) naso-frontal suture, a flat, tabular dorsal surface of the involucrum, and an anterior process of the periotic with a dorsally deflected anterodorsal angle.

## 4. Description

### 4.1 Skull

#### 4.1.1 Cranium

**Figure 2.**
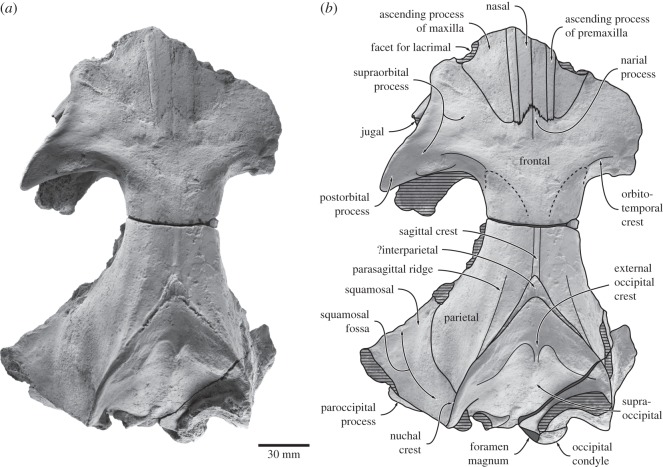
Cranium of *Fucaia buelli* in dorsal view: (*a*) photograph and (*b*) line drawing.

**Figure 3.**
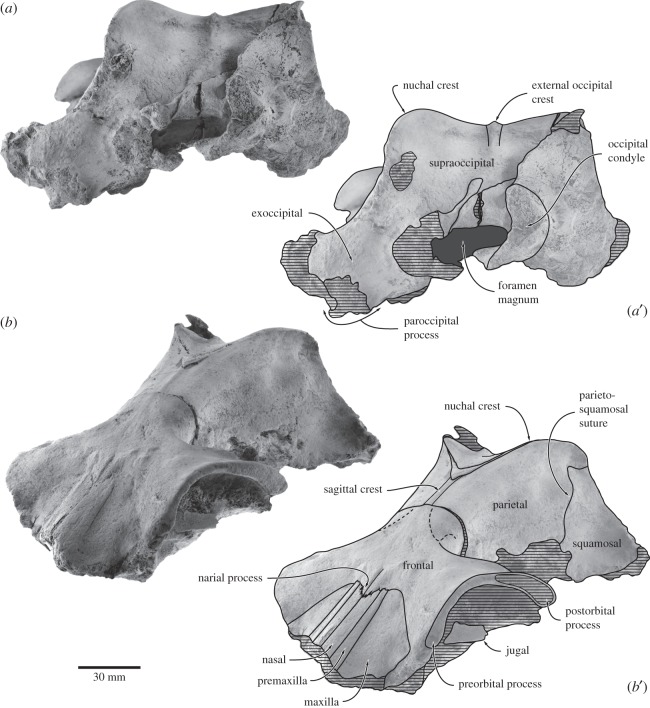
Cranium of *Fucaia buelli* in (*a*) posterior and (*b*) anterolateral view. (*a*,*b*) photographs and (*a*^′^,*b*^′^) line drawings.

**Figure 4.**
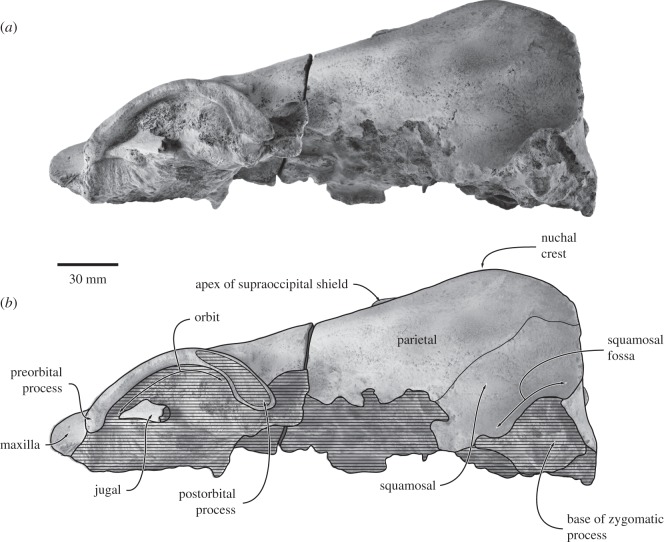
Cranium of *Fucaia buelli* in lateral view. (*a*) Photograph and (*b*) line drawing.

**Figure 5.**
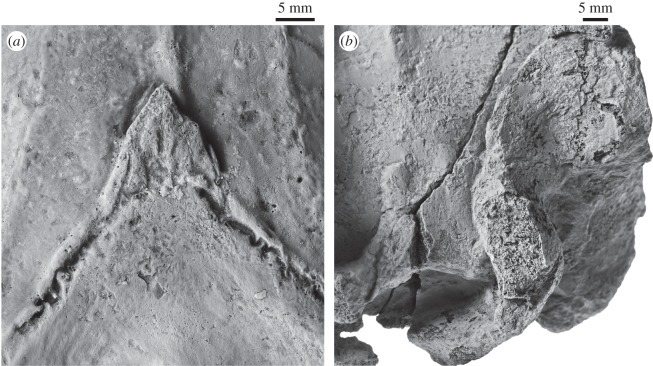
Details of the cranium of *Fucaia buelli*. (*a*) Apex of supraoccipital shield in dorsal view, showing the possible interparietal; (*b*) displaced right exoccipital revealing an unfused planar elongate surface of the supra-exoccipital suture immediately anterior to the eroded upper margin of the right occipital condyle.

**Figure 6.**
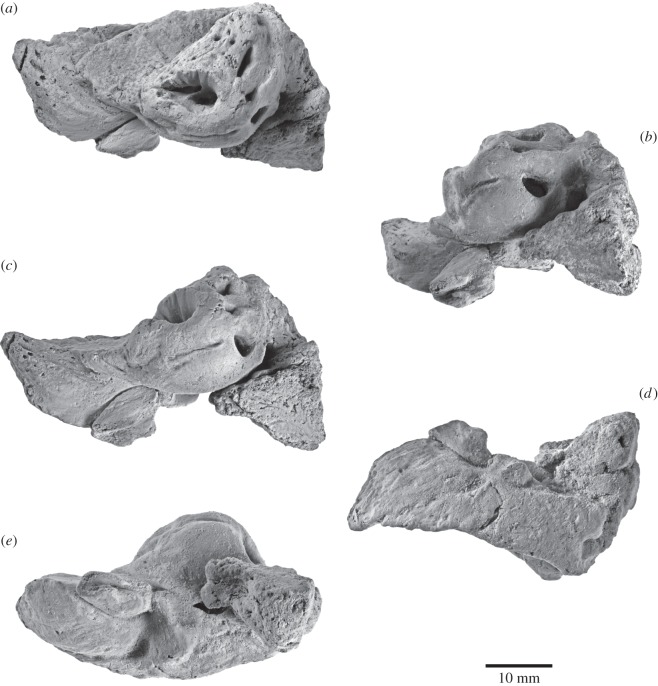
Right periotic of *Fucaia buelli*. Specimen shown in (*a*) dorsal, (*b*) posteromedial, (*c*) medial, (*d*) lateral and (*e*) ventral view. (*a*–*e*) Photographs and (*a*^′^–*e*^′^) line drawings.

The preserved portion of the cranium includes the dorsal and lateral surfaces of the braincase, the entire left and part of the right supraorbital process, both periotics, the right tympanic bulla and malleus, both stapes, and the left incus (figures 2–6; tables 1 and 2). The left occipital condyle and the basicranium are severely eroded, with the latter preserving effectively no detail at all. Much of the outer surface of the tympanic bulla, including the lateral portion of the sigmoid process, broke away during collection or initial preparation, and is only preserved as a thin layer of bone embedded in a separate lump of matrix. The right exoccipital and a small part of the supraoccipital were broken during burial, and are displaced relative to the rest of the skull. Except for a seemingly somewhat loose connection between the anterior portion of the supraoccipital and the parietals and a vestige of the supra-exoccipital suture (see below), the cranial sutures are largely closed. As in virtually all other archaic cetaceans, the temporal fossa is longer anteroposteriorly than wide transversely in dorsal view. As in *F. goedertorum*, the intertemporal region is robust, yet anteroposteriorly elongate.

**Figure:**
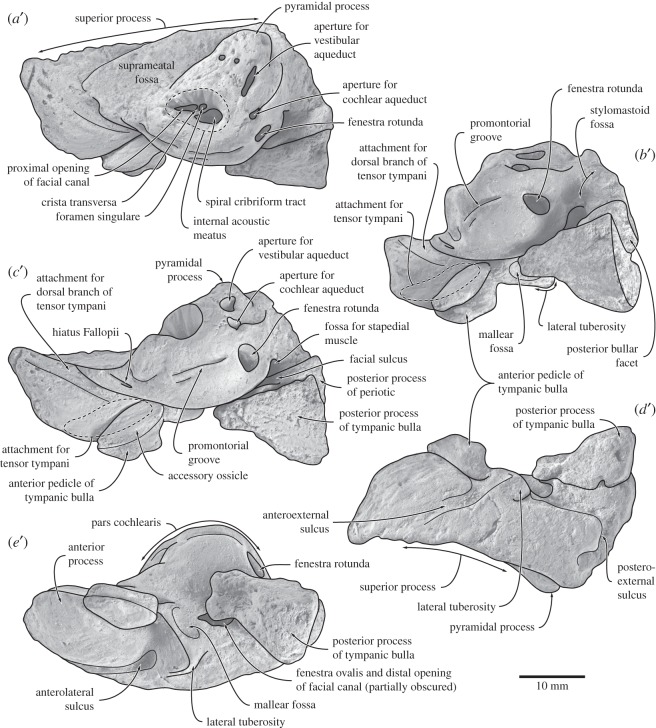

**Table 1.** Measurements (in millimetres) of the holotype skull of *Fucaia buelli* (UWBM 84024).

cranial length, from anterolateral inflection of supraorbital process of frontal to posterior surface of occipital condyle	235.0^*a*^
maximum preorbital width	122.88^*b*^
maximum postorbital width	184.68^*b*^
supraorbital width across middle of the orbits	133.56^*b*^
preserved diameter of left orbit, from apex of preorbital process of frontal to preserved apex of postorbital process	69.78
length of left nasal, as preserved	62.0
maximum width of left nasal	17.04
distance between posterior edge of nasals and apex of supraoccipital	106.19
maximum width of the parietal within the temporal fossa	119.38^*b*^
maximum bicondylar width	59.22^*a*^
maximum height of right occipital condyle	36.93^*a*^

^*a*^Estimated measurement.

^*b*^The measurements are made from one side to the midline of the skull and multiplied by two.

**Table 2.** Measurements (in millimetres) of the ear bones of *Fucaia buelli*. Holotype, UWBM 84024.

right tympanic bulla	
maximum length in dorsal view	55.78
maximum length of the tympanic cavity	47.80
height of inner posterior prominence, measured between anterior border of inner posterior pedicle and the transverse ridge crossing the interprominential notch	19.57
width of tympanic cavity at the level of the lateral furrow, between inner face of outer lip and lateral face of involucrum, avoiding the involucral incisure	ca 13.5
distance between dorsal margin of lateral furrow and apex of tympanic bulla, measured horizontally	27.89
right periotic	
maximum length in dorsal view	44.72
maximum diameter of internal acoustic meatus	9.88
maximum diameter of aperture for cochlear aqueduct	2.64
maximum diameter of aperture for vestibular aqueduct	5.53
maximum diameter of fenestra rotunda	4.54
maximum diameter of mallear fossa	6.66
maximum length of fovea epitubaria	10.89
height of the anterior keel	18.10
distance between anteroventral angle and tip of lateral tuberosity	16.50
distance between anteroventral angle and caudal tympanic process	27.56
right malleus	
maximum dorsoventral length (excluding anterior process)	9.00
maximum transverse width (excluding anterior process)	4.50
left incus	
maximum width at the level of the incudo-mallear joint	4.50

#### 4.1.2 Maxilla, premaxilla and nasal

Only the ascending processes of the maxillae and premaxillae are preserved, and, along with the nasals, extend posteriorly roughly to the level of the central portion of the orbit. The fronto-maxillary suture is barely apparent, but can be traced along almost its entire preserved length. In dorsal view (figure 2), the ascending process of the maxilla is triangular and terminates at the same level as the premaxilla. Just medial to the preorbital process of the frontal, the lateral border of the ascending process of the maxilla curves inwards and forms a broad embayment for the now-lost lacrimal. The ascending process of the premaxilla is nearly parallel-sided and only gently tapers towards its posterior end. Compared with the ascending process of the maxilla, the premaxilla is extremely slender (approx. 5 mm, compared with 29+ mm for the maxilla at its widest point). Nevertheless, it still forms a clear contact with the frontal and thus completely separates the maxilla from the nasal. In lateral view, the faint suture between the premaxilla and the nasal runs slightly dorsal to the suture between the maxilla and premaxilla. The nasal is relatively broad and anteroposteriorly elongate, although its exact length remains unknown because of anterior breakage on both sides. The anterior portion is transversely arched, whereas its posterior end is relatively planar. Posteriorly, the nasal tapers towards the lateral side and is separated from its counterpart by a triangular narial process of the frontal.

#### 4.1.3 Frontal

In dorsal view, the frontals are clearly exposed along a *ca* 85.0 mm long section of the skull vertex and contact the parietals along a barely distinguishable, seemingly V-shaped suture (see comment below about possible interparietal). Anterior to the intertemporal constriction, each frontal extends laterally to form the triangular supraorbital process, the anteromedial portion of which is overlain by the ascending processes of the maxilla and premaxilla. As in other mysticetes, the preorbital process remains distinct in dorsal view and is not covered by the maxilla. The dorsal rim of the large orbit is gently curved and oriented at an angle of *ca* 25 degrees relative to the sagittal plane. The preorbital process is small, smoothly curved and does not project from the outline of the orbit in dorsal view. By contrast, the postorbital process is well developed and projects posterolaterally far beyond the level of the preorbital process.

Near its posterior border, and running nearly parallel to it, the dorsal surface of the supraorbital process bears a well-developed orbitotemporal crest delimiting a clearly defined fossa for the origin of the temporal muscle. Laterally, the orbitotemporal crest fades away as it approaches the postorbital process, while a secondary, slightly more posteroventrally located ridge develops close to the posterior border of the supraorbital process; medially, the orbitotemporal crest curves backwards and terminates just anterior to the fronto-parietal suture. There are no obvious foramina on the dorsal surface of either supraorbital, although their absence could at least partially be a result of surface erosion. In anterior view (figure 3), the supraorbital process is essentially horizontal and only slightly descends from the level of vertex. In lateral view (figure 4), the orbit is distinctly arched. Unlike the robust postorbital process, the preorbital process is thin dorsoventrally and barely distinguishable from the more central portion of the orbital rim. The lateral surface of the postorbital process is planar, possibly as a result of erosion. The medial wall of the temporal fossa is too eroded for the fronto-parietal suture to be traced, although comparisons with structurally similar taxa, such as *F. goedertorum*, suggest that the frontal probably contributed to the formation of the lateral skull wall.

#### 4.1.4 Parietal

Each parietal is broadly exposed on the skull vertex and forms most of the lateral and dorsal walls of the braincase. The dorsal surface of the parietals is flattened, but bears a low (less than 1.5 mm) sagittal crest running from the apex of the supraoccipital towards the posteriormost point of the fronto-parietal suture (figure 2). Lateral to the sagittal crest, there is a separate, parasagittal ridge originating from the central portion of the nuchal crest and running anteriorly somewhat past the level of the apex of the supraoccipital shield. This ridge in turn is paralleled by a second, considerably fainter ridge, which disappears posteriorly before it reaches the nuchal crest. In lateral view, the dorsal surface of the parietal remains in a straight line with the frontal and rises only gently towards the supraoccipital, thus resulting in a low dorsal skull profile. Posteriorly, the parietal contacts the squamosal along a ridge-like suture descending anteroventrally from the posterior quarter of the nuchal crest. The ventral portions of both parietals are lost.

#### 4.1.5 Supraoccipital

In dorsal view, the supraoccipital is triangular with convex lateral borders (figure 2). Anteriorly, the supraoccipital terminates at, or just anterior to, the level of the anterior border of the squamosal fossa, and well posterior to the level of the postorbital process. It is possible that the small projecting apex of the supraoccipital shield is actually interparietal, given its bounding complex fine sutures (figure 5*a*). An external occipital crest originates *ca* 40.0 mm posterior to the apex of the supraoccipital, but fades away before it reaches the foramen magnum. Between the external occipital crest and the nuchal crest, there is a platform- or shelf-like, almost horizontal tuberosity extending along approximately two-thirds of each lateral margin of the supraoccipital. Posterior and medial to the tuberosity, the surface of the supraoccipital descends steeply towards the foramen magnum. In anterior view, the nuchal crest is distinct, but does not overhang the lateral skull wall (figure 3*b*). Along its anteriormost portion, the nuchal crest is nearly flush with the flattened skull vertex, except for a small tuberosity on the dorsal surface of the parietals. In lateral view, the nuchal crest is convex dorsally and obscures the dorsal surface of the supraoccipital (figure 4). In posterior view, the dorsal surface of the supraoccipital is clearly concave and bounded laterally by the relatively high nuchal crest (figure 3*a*).

#### 4.1.6 Exoccipital and basioccipital

The suture between the exoccipital and the supraoccipital is indistinguishable on the left, but is apparent as a displaced, *ca* 14.4 mm long vestige above and continuous with the displaced right condyle (figure 5*b*). The occipital condyles are heavily damaged and seemingly asymmetrical, thus implying a degree of post-mortem distortion (figure 3*a*). The exact shape of the foramen magnum is uncertain, but it appears to have been elliptical and transversely wider than high. In posterior view, the dorsal border of the exoccipital has a slight dorsal condyloid fossa; the ventral surface is too damaged to be sure of a ventral condyloid fossa. Lateral to the occipital condyle, the paroccipital process descends ventrally to the level of the basioccipital crest, or just below. The jugular notch is lost. The basicranium is heavily eroded, with only the left basioccipital crest remaining intact. The latter is somewhat thickened transversely and oriented slightly posterolaterally.

#### 4.1.7 Squamosal

Both of the squamosals are poorly preserved and have lost their zygomatic processes. The parieto-squamosal suture is elevated into a low ridge. There is no squamosal cleft. In dorsal view, the posterolateral portion of the squamosal, which originally would have given rise to the postglenoid process, forms a distinct angle with the lateral border of the exoccipital. The length of the squamosal fossa is uncertain, but it probably terminated at or just posterior to the level of the apex of the supraoccipital. The nuchal crest does not project posteriorly beyond the level of the occipital condyle. Given the proportions of the cranium and its overall resemblance to that of *F. goedertorum*, it seems unlikely—though not impossible—that the anterior tip of the zygomatic process would have reached the level of the postorbital process. The roof of the right external meatus is a sub-cylindrical transverse groove 13+ mm long.

#### 4.1.8 Periotic

The right periotic is isolated, whereas the left periotic is preserved *in situ*. In dorsal view (figure 6*a*), the anterior process is relatively short and separated from the pars cochlearis by a clearly defined incisure. The dorsolateral border of the periotic is formed by a low superior process, which runs posteriorly as far as the posterior border of the pars cochlearis. Medial to the superior process, the suprameatal area is moderately concave and shows no sign of hypertrophy. Posteriorly, the suprameatal fossa is bordered by a well-developed, tall pyramidal process, which is clearly separated from both the fossa and the adjacent superior process by a deep, longitudinal sulcus. We use the term pyramidal process *sensu* Luo & Gingerich [25] provisionally, noting that the homology of this feature in Mysticeti has been questioned [14]. The medial surface of the pyramidal process is pitted, with two relatively large openings being located just in front of the aperture for the vestibular aqueduct.

The internal acoustic meatus (including the proximal opening of the facial canal) is broadly teardrop-shaped and bears a series of dorsoventral grooves on its lateral wall. Unusually, the transverse crest separating the proximal opening of the facial canal from the dorsal vestibular area is poorly developed, to the point that it is lower than the crest separating the foramen singulare from the spiral cribriform tract and the area cribrosa media (figure 6*a*). The proximal opening of the facial canal is slit-like, and much smaller than the dorsal vestibular area. The foramen singulare is elliptical, and located directly posterior to the proximal opening of the facial canal. The spiral cribriform tract and the area cribrosa media are essentially confluent and separated from the more laterally located foramen singulare by a distinct crest. The aperture for the vestibular aqueduct is slit-like and does not overlap (anteroposteriorly) with the much smaller aperture for the cochlear aqueduct. The latter is roughly circular and laterally opens into a distinct sulcus running transversely across the posterior face of the pars cochlearis. The posterior process is short anteroposteriorly with a rhomboidal bullar facet and connected to the body of the periotic via a transversely robust neck (figure 6*b*).

In medial view (figure 6*c*), the anterior process is sickle-shaped, with a convex anterior keel and a dorsally deflected anterodorsal angle. The ventral border of the anterior process is obscured by the accessory ossicle of the tympanic bulla, which sits in the fovea epitubaria. Except for its posteriormost portion, which is confluent with the rim of the mallear fossa, the accessory ossicle is not fused to the periotic (figure 6*d*). Ventrally, the accessory ossicle gives rise to the broken base of the anteroposteriorly elongate anterior pedicle of the bulla. Dorsomedially, the accessory ossicle and a portion of the medial side of the anterior process form the site of origin of the main portion of the *m. tensor tympani*. Further dorsally, a second branch of this muscle may have originated from a deep, anterodorsally directed sulcus located just anterior to the pars cochlearis (figure 6*c*). Just posterior to this sulcus is a narrow, slit-like opening, probably the hiatus Fallopii. The pars cochlearis rises dorsally above the surface of the suprameatal area. Both the anteromedial and posterolateral portions of the rim of the internal acoustic meatus are distinctly convex. Posterolaterally, the rim of the internal acoustic meatus merges with the robust pyramidal process, which marks the dorsalmost point of the periotic. Medial to the pyramidal process, the apertures for the vestibular and cochlear aqueducts open dorsomedially, one ventral to the other. Posterior to the pyramidal process, the sulcus running along the posterior face of the pars cochlearis is evident as a distinct shelf and clearly separated from the more posteroventrally located stylomastoid fossa (figure 6*b*,*c*).

The fenestra rotunda is relatively large, separated from the aperture for the cochlear aqueduct and flush with the posteromedial border of the pars cochlearis. The caudal tympanic process is effectively absent and hence does not approach the crista parotica. Anterior to the fenestra rotunda there is a clearly defined promontorial groove, the anterior third of which is paralleled by a second, slightly more ventrally located sulcus. Ventral to the proximal opening of the facial canal, the promontorial groove is replaced by a broader depression. The latter in turn gives rise to a sulcus, which turns dorsally and terminates somewhat anterior to the internal acoustic meatus. The posterior process of the periotic is rather short and not fused to that of the tympanic bulla. In figure 6, the posterior process of the bulla is still attached to the right periotic but has been displaced anteriorly post-mortem along the posterior bullar facet, so that its apex is now directly juxtaposed to the ventral margin of the pars cochlearis.

In lateral view, the surface of the periotic is mostly smooth (figure 6*d*). Just anterior to the lateral tuberosity, a well-developed anteroexternal sulcus runs anterodorsally along the anterior process. Dorsolateral to the base of the posterior process there is a posteroexternal sulcus, which may carry the posteroexternal foramen. In ventral view (figure 6*e*), the anterior process is somewhat thickened transversely. There is no anterior bullar facet. Along its lateral portion, the anterior process is separated from the body of the periotic by a deep pit (distinct from the more medially located fovea epitubaria), which slightly excavates the anterior face of the lateral tuberosity. The latter is transversely short and anteroposteriorly compressed, with its ventralmost portion in particular being pinched between the aforementioned pit anteriorly and the well-defined mallear fossa posteriorly. The outline of the pars cochlearis is rounded, but bears a distinct anteromedial ridge that runs towards the anterior process. The fenestra ovalis, distal opening of the facial canal, stapedial muscle fossa and most of the posterior bullar facet are obscured by the stapes and the anteriorly displaced posterior process of the tympanic bulla.

#### 4.1.9 Tympanic bulla

**Figure 7.**
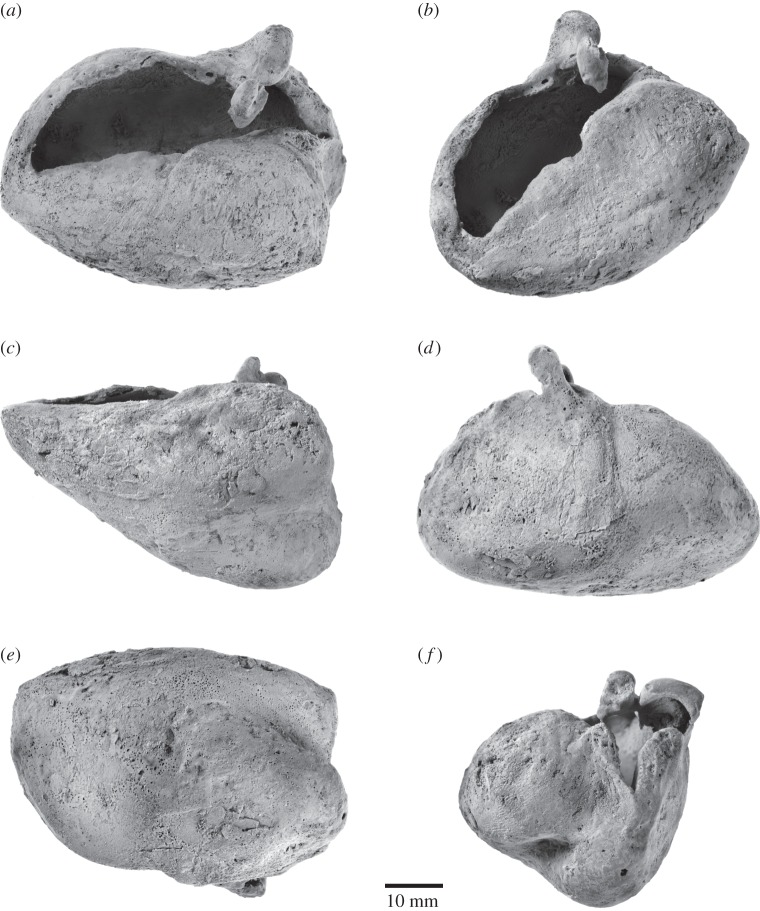
Right tympanic bulla of *Fucaia buelli*. Specimen shown in (*a*) dorsal, (*b*) anteromedial, (*c*) medial, (*d*) lateral, (*e*) ventral and (*f*) posterior view. (*a*–*f*) Photographs and (*a*^′^–*f*^′^) line drawings.

The right tympanic bulla is relatively well preserved, although part of the bone surface has disappeared. In dorsal view (figure 7*a*), the outer lip is distinctly convex. The sigmoid process is located far posteriorly, thus partially obscuring the conical process. The anterior surface of the sigmoid process slopes anteroventrally towards the elongate mallear ridge. Anterior to this ridge, the sulcus for the chorda tympani runs horizontally along the inner wall of the outer lip, but is too damaged to determine its exact length or shape. Posterior to the conical process, the inner and outer posterior pedicles are separated by a deeply incised elliptical foramen. The anteriormost point of the involucral ridge is obscure, but seems to form the anteriormost point of the tympanic bulla. The involucrum is relatively wide transversely and developed as a shelf medially bordering the tympanic cavity. There seem to be no major transverse creases on the dorsal surface of the involucrum, although this may partially be an artefact of bone surface erosion. A step-like, centrally positioned *involucral incisure* (new term) subdivides the involucrum into a thicker posterior portion and a narrower anterior one (figure 7*b*). Internally, the involucral incisure roughly lines up with a transverse ridge running from the inside of the involucrum towards the floor of the tympanic cavity.

**Figure:**
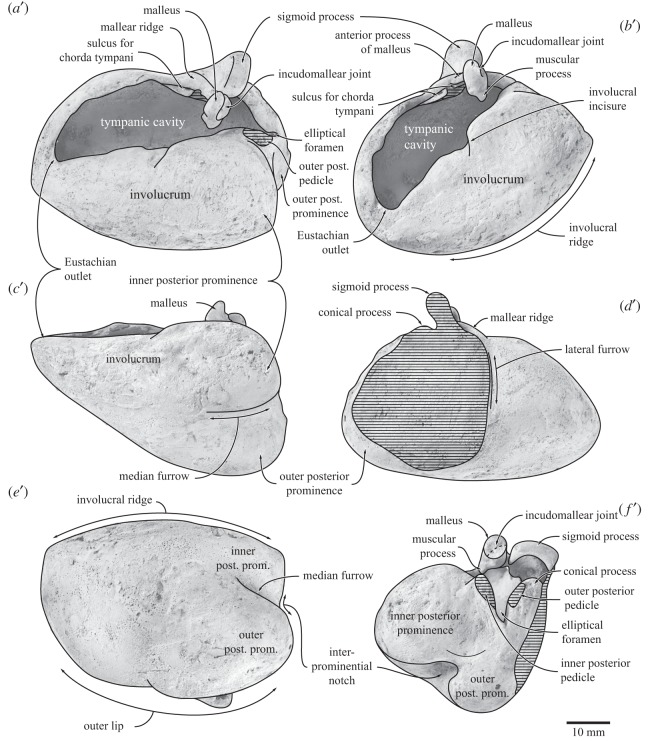

In medial view (figure 7*c*), the tympanic bulla strongly tapers towards its anterior end. The dorsal surface of the involucrum is flattened, particularly along its anterior half. The outer posterior prominence (=lateral lobe) extends further ventrally than the inner one (=medial lobe), and is separated from the latter by a well-developed median furrow. In lateral view (figure 7*d*), a shallow lateral furrow originates anterior to the mallear ridge and runs vertically towards the ventral portion of the outer posterior prominence. Posterior to the lateral furrow, the outer posterior prominence is somewhat bulbous and ventrally convex, whereas more anteriorly the ventral surface of the bulla is flattened anteroposteriorly and transversely rounded. The sigmoid process lies above the anterior margin of the conical process. Its base is damaged, with the profile of the sigmoid cleft uncertain. What remains is consistent with a cleft descending at least obliquely forward, but whether there was a horizontal ventral margin, as in archaeocetes, odontocetes and other archaic mysticetes (e.g. [6,26,27]), is uncertain. The conical process is relatively small, convex dorsally, and located directly below the posterior margin of the sigmoid process.

In ventral view (figure 7*e*), the outline of the tympanic bulla is obliquely truncated by its posterolaterally directed anterior border. The medial border of the bulla, corresponding to the involucral ridge, is only slightly curved and posteriorly forms a right angle with a transverse crest running along the posterior face of the medial lobe. By contrast, the posterior portion of the lateral border of the bulla is distinctly convex and bulges outwards posterior to the lateral furrow. The inner and outer posterior prominences are separated from each other by the median furrow and interprominential notch. The median furrow is oriented slightly anteromedially, deeply concave and becomes indistinct roughly at the level of the sigmoid process.

In posterior view (figure 7*f*), the outer posterior prominence is markedly narrower than its inner counterpart. The interprominential notch is deep, rounded and skewed towards the outer prominence, reflecting the anteromedial orientation of the median furrow. At the level of the interprominential notch, a transverse crest runs along the posterior face of the inner prominence and indistinctly on to the outer one. Dorsally, the interprominential notch is separated from the inner posterior pedicle of the tympanic bulla by a shallow depression. The inner and outer posterior pedicles are located on either side of the elliptical foramen, with the inner pedicle being more robust. As far as can be told, the sigmoid process is squared in outline.

#### 4.1.10 Auditory ossicles

The right malleus and both stapes are preserved *in situ*, whereas the left incus was found in isolation next to the hyoid bones. The elongate anterior process of the malleus arises from the mallear ridge of the tympanic bulla. Anteromedially, the anterior process conducts the sulcus for the chorda tympani from the outer lip of the bulla towards the head of the malleus (figure 7*b*). The main body of the malleus is robust, dorsoventrally elongate and positioned close to the dorsomedial corner of the sigmoid process, but not confluent with the latter as in most living balaenopterids (figure 7*b*, *f*). As preserved, the sigmoid process and malleus are separated from each other by a narrow cleft, although the latter may be a result of post-mortem displacement.

In anterior view, the head of the malleus is sub-spherical and rises well above the level of the robust anterior process. The eroded incudal facets appear to be roughly perpendicular to each other; they are too poorly preserved to be certain about their shape and size, but the anterior facet seems to be the larger of the two and presumably is oriented posteriorly, whereas the smaller posterior facet points dorsomedially. The hook-like, ventrally placed manubrium curves slightly laterally and forms the insertion for the tympanic ligament. The muscular process is a transverse pit on the anterior face of the malleus for the insertion of the tendon of the *m. tensor tympani* (figure 7*f*). The poorly preserved left incus is conical, with a robust body bearing the articular facets for the malleus, as well as the dorsomedially curved crus longum and the slender crus breve—both of which are damaged. The stapes is rod-shaped, with its base, or footplate, being wider than its head. The stapedial foramen is patent and relatively well developed. The head of the stapes is offset from the foramen by a robust neck.

#### 4.1.11 Mandible

**Figure 8.**
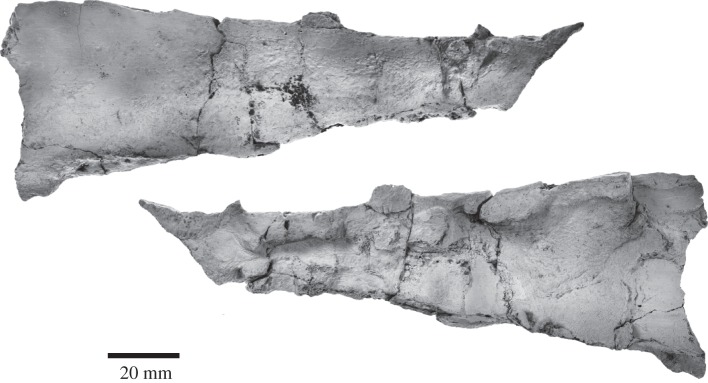
Fragment of right mandible of *Fucaia buelli*.

Only a fragment (length: 162.0 mm, dorsoventral height ranging from 21.0 mm anteriorly to 56.7 mm posteriorly) of what appears to be the right mandible is preserved (figure 8). There are no clear alveoli and no mental foramina. The fragment is smooth, inflated laterally and concave medially, but there is not enough preserved to tell which part of the mandible is represented.

#### 4.1.12 Dentition

**Figure 9.**
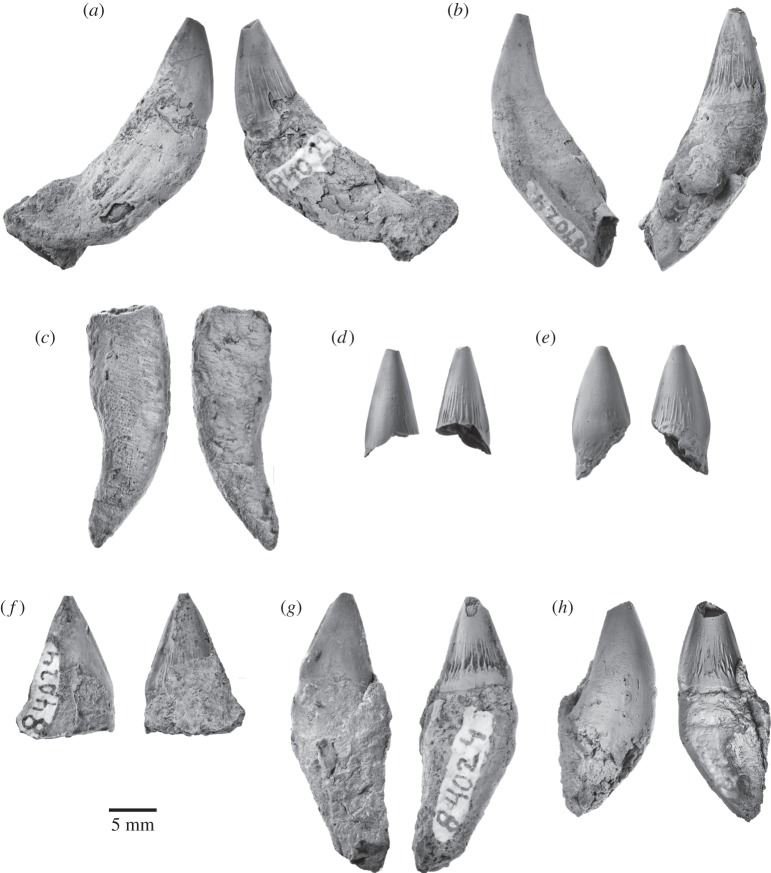
Anterior teeth of *Fucaia buelli* in labial (left) and lingual (right) view. (*a*–*e*) Presumed incisors; specimen shown in (*c*) preserves only the root; (*f*) potential first premolar; (*g*) presumed left P1; (*h*) presumed left p1.

**Figure 10.**
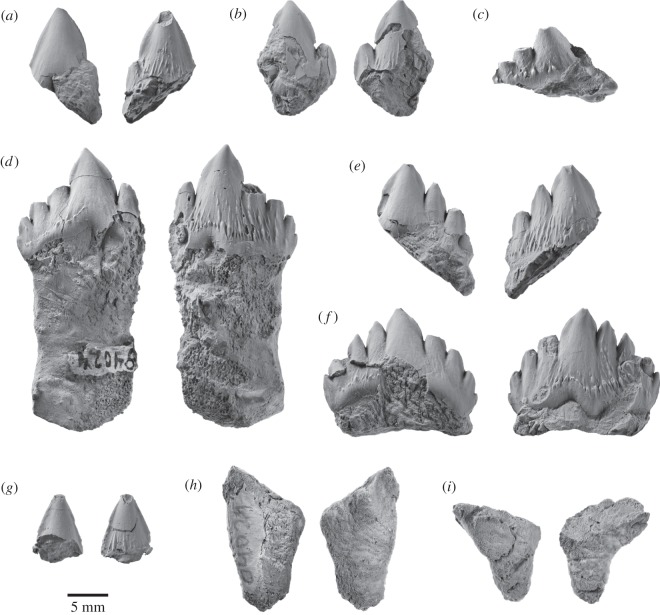
Postcanine teeth of *Fucaia buelli*. (*a*,*b*) Presumed P2/p2; (*c*) presumed posterior left upper molar in labial view; (*d*) presumed left P3; (*e*) presumed right P3; (*f*) presumed left M1; (*g*) presumed main denticle of postcanine, position unknown—possibly a first premolar; (*h*,*i*) isolated roots presumed to represent postcanines (post-P2/p2). All teeth except (*c*) are shown in labial (left) and lingual (right) view; the orientations of the root fragments (*h*,*i*) are uncertain.

**Figure 11.**
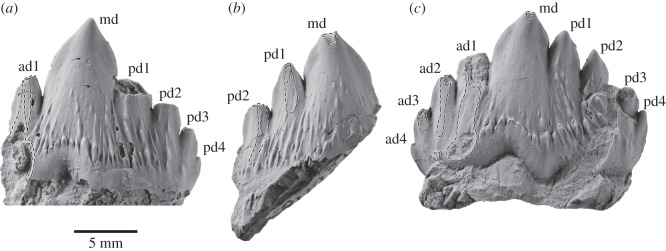
Details of attritional and abrasive tooth wear. (*a*) Left P3; (*b*) right P3, (*c*) left M1, all in lingual view.

The specimen preserves 17 isolated teeth (figures 9–11), six of which bear accessory denticles and thus represent premolars or molars (figure 10*a*–*f*). A further three specimens probably represent isolated cusps or roots that originally belonged to a denticulate tooth (figure 10*g*–*i*). All the tooth crowns are lingually curved in mesial or distal view—markedly so in the case of the single-cusped teeth, less or barely so in the case of the denticulate ones. In addition, they show a consistent difference between the lingual and labial enamel ornament: whereas the labial faces are smooth with occasional faint, sub-vertical elongate nodules or ridges near the crown base, the lingual faces present prominent, often sharp-crested ridges that may extend to the centre of the crown and even rise on to the denticles. Individual cusps and denticles bear anterior and posterior carinae, which are particularly well developed and markedly convex on the main denticle of the postcanines. Cingula on the anterior, single-cusped teeth only occur in the form of a largely smooth band of enamel running along the lingual crown base. On the more-posterior teeth, the cingula are better developed, taking the shape of a series of nodular papillae lingually and a faint band of elongate nodules labially (figure 10*c*,*d*,*f*). In addition, at least some of these teeth possess one minor, cingular nodule at both the anterior and posterior extremities of the crown; however, there is no continuous cingular shelf as seen in basilosaurids and (lingually only) *Morawanocetus*.

Because of the loss of the rostrum and mandibles, there is no information on tooth number, position or the presence of diastemata, which makes the identification of individual elements provisional. Crown symmetry, number and position of denticles, and occlusal patterns are used to identify tooth position and quadrant, by analogy with basilosaurid archaeocetes. The incisors and canines (figure 9) and, possibly, the first premolar have a simple sub-conical crown with a single cusp. The shape of the crown varies from tall and slender (incisors; figure 9*a*–*e*) to somewhat more broad-based and robust (canines or first premolars; figure 9*f*–*h*). Two teeth with laterally compressed crowns and slightly crenulated keel bases may be left P1 (slightly more posteriorly curved; figure 9*g*) and left p1 (figure 9*h*). Three of the incisors preserve single roots, both of which are lingually curved (figure 9*a*–c); the same holds true for the presumed first premolars, although in this case the roots are straighter and considerably more bulbous (figure 9*g*,*h*). None of these anterior teeth show clear attritional or abrasive wear.

By analogy with Basilosauridae, P2 and more-posterior teeth would have one or more accessory denticles at least on the posterior keel, and for most also on the anterior keel. The term denticle is used here because of uncertain homology with the cusps of other mammals. To help describe structure and tooth wear below, we code denticles (**d**) as main (**md**), anterior (**a**, with numbering away from the main denticle: ad1, ad2, etc.) or posterior (**p**, numbered away from the main denticle: pd1, pd2, etc.). The most basal projections could be described as small denticles or, equally, nodules. For *Fucaia*, it is not known whether there are anterior denticles in m1–3, or whether, like Basilosauridae, anterior denticles are absent. *Fucaia* is otherwise presumed to be basilosaurid-like, with P3–M2 and p3–p4 having both anterior and posterior denticles.

Only four of the denticulate teeth retain the main portion of the crown (figure 10*c*–*f*). In general, the latter is relatively low and elongate, in contrast with the more triangular premolars and lower molars of basilosaurids and the taller, more conical teeth of mammalodontids and *Aetiocetus* [3,6,9,28]. Comparatively low crowns also occur in basilosaurids (upper molars only), *Llanocetus* and *Morawanocetus*, but those of *F. buelli* are considerably less robust, with more delicate, closely spaced sub-vertical denticles and no sign of transverse inflation [1,10]. Two crowns lacking roots, but laterally compressed and with one presumed pd, may be P2 and p2 (figure 10*a*,*b*), but which is upper and lower is uncertain. One of these crowns (figure 10*a*) shows attritional wear towards the base of the anterior keel, but without exposing dentine.

Three other denticulate teeth show attritional and/or abrasive wear. figure 10*d* shows a presumed left P3 with md, ad1, pd1, pd2, pd3 and a basal nodule (pd4); it is not known if the matrix contains any roots. The lingual face has prominent sub-parallel and sub-vertical, sharp enamel ridges, above a row of nodules forming an incipient cingulum; the labial face has only minor ornament. The crown base is inflated lingually below the junction of md–pd1, forming a bulge that in other heterodont cetaceans has been interpreted as a protocone remnant [29,30]; whether there was an associated third root is unknown. In addition to the protocone bulge, an upper left position is also indicated by the slightly lingually curved crown and attritional wear patterns present lingually but not labially (it is assumed that, as in basilosaurids, the upper cheek teeth occluded lateral (labial) to the lower cheek teeth). The worn apex of ad1 forms a window in the enamel, with labially curved, irregularly worn enamel surfaces consistent with abrasion (figure 11*a*); there is no consistent pattern of microwear to the eye (Zeiss Stemi 2000C binocular microscope, 50×). Lingually, immediately below the apex, ad1 carries two barely separable, planar sub-vertical attritional wear facets, extending to the crown base and forming a prominent window in the enamel (figure 11*a*). The facets truncate enamel ridges, and under reflected light (50×) show strong vertical microwear.

A presumed right P3 (figure 10*e*) is less complete than but otherwise mirrors the left P3 of figure 10*d*. The roots and anterior denticles are lost. The slightly inflated crown above the break for the roots is consistent with a protocone remnant. Apical and lingual wear is evident (figure 11*b*). The md, pd1 and pd2 apices have polished worn enamel surfaces curving lingually, with dentine exposed in windows on md and pd2. Two attritional wear facets (two planes at a slight angle) on pd1 are partly rounded by presumed abrasion toward the pd1 apex, but more polished basally; similarly, pd2 has two attritional wear facets at a slight angle, more rounded (abraded?) apically, and more mirror-polished basally.

A low denticulate crown (figure 10*f*) with slight asymmetry (shorter presumed anterior keel than posterior keel) is interpreted as a left M1. There is no lingual inflation that might indicate a protocone remnant. The md is canted slightly posteriorly, and ad1, ad2, ad3 and small ad4 descend more steeply than pd1, pd2, pd3 and pd4. Rounded apical wear on md (with a tiny window of dentine), ad2 and ad3 is presumably abrasive. Fine striations descending from near the apex of md form a rounded plane which may have been attritional with superimposed abrasive wear. On ad1, two barely separable polished facets descend to below the cingulum, exposing dentine. On ad2, a polished facet descends below the denticle base; a more-basal cingular nodule also has a polished facet. On ad3, two polished facets mark two separate planes toward the apex (figure 11*c*).

The last of the denticulate teeth is still partially encased in matrix, attached to the basihyal, and consists of the labial face of a low, elongate crown preserving md and at least three posterior denticles (figure 10*c*). The denticle apices are worn off; rounded enamel surfaces seen on two denticles at 50× magnification suggest abrasive wear. There is no obvious attritional wear; the lingual face, which might otherwise show such wear, is missing. Based on its shape, the tooth probably represents a posterior left upper molar. None of the denticulate teeth preserve any roots, but there are two sets of presumed isolated roots that, based on their size, probably represent postcanines (figure 10*h*,*i*). In the more intact of these specimens, the roots taper and converge ventrally, and are closely apposed or even fused along their entire length (figure 10*h*).

#### 4.1.13 Hyoid apparatus

**Figure 12.**
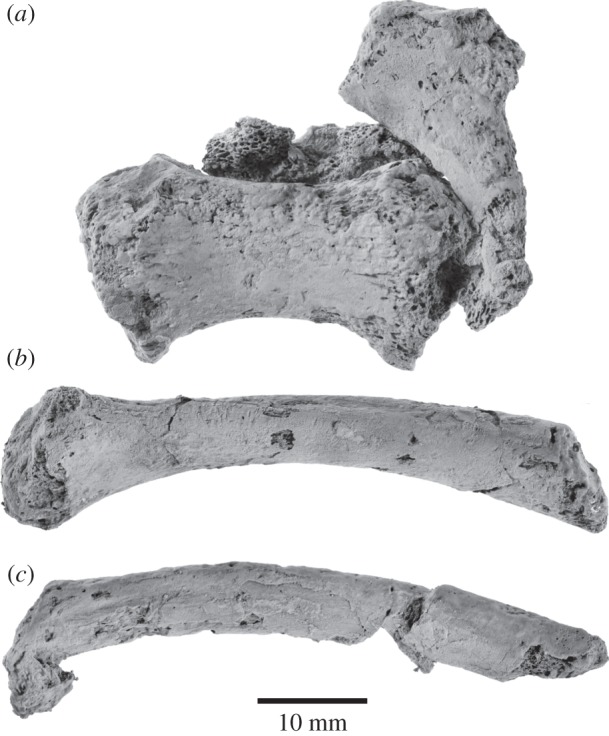
Hyoid apparatus of *Fucaia buelli*. (*a*) Basihyal, with the partial left thyrohyal (top right) and other bone fragments still attached; (*b*) right thyrohyal; (*c*) right stylohyal. All in dorsal view.

The preserved portion of the hyoid apparatus includes the basihyal, the right thyrohyal and stylohyal, and the proximal portion of the left thyrohyal (figure 12 and table 3). The basihyal is dumbbell-shaped and dorsoventrally flattened. Laterally it bears a relatively large, posterolaterally pointing facet for articulation with the thyrohyal. The point of articulation with the probably unossified ceratohyal [31] is smaller and less well defined. The thyrohyal is elongate, robust, moderately curved and expanded both proximally (where it articulates with the basihyal) and distally. The central portion of this bone is more or less circular in cross section, unlike the more flattened thyrohyal of many balaenopteroids. The stylohyal resembles the thyrohyal in terms of its length, but is much more gracile and of nearly equal width throughout.

**Table 3.** Measurements (in millimetres) of the hyoid elements of *Fucaia buelli*. Holotype, UWBM 84024.

maximum width of basihyal	39.05
maximum anteroposterior length of basihyal	18.82
maximum dorsoventral height of basihyal	15.36
anteroposterior length of body of basihyal	12.22
dorsoventral height of body of basihyal	7.13
length of thyrohyal, as preserved	57.04
maximum diameter of proximal end of thyrohyal	14.31
maximum diameter of body of thyrohyal	8.36
length of stylohyal, as preserved	61.32
maximum diameter of proximal end of stylohyal	10.31
maximum diameter of body of stylohyal	7.90

### 4.2 Postcranial skeleton

The specimen comprises 20 vertebrae (table 4), including seven unfused cervicals, 10 thoracics and three lumbars. Except for some of the thoracics, all the vertebrae appear to have both epiphyses fused to the centra.

**Table 4.** Measurements (in millimetres) of the vertebrae of *Fucaia buelli*. Holotype, UWBM 84024. Dimensions are maxima, in dorsoventral, anteroposterior or mediolateral planes, unless stated. For bilateral measurements, the more complete side is used, as stated.

atlas	
height, tip of neural spine to ventral margin of hypapophysis	74.89
transverse width, including transverse processes	107.75
anteroposterior length, measured along ventral border	19.79
height of neural canal *sensu lato*, including fovea dentis	35.09
width of neural canal	36.03
height of articular facet for occipital condyle, right	43.07
width of articular facet for occipital condyle, left	20.09
bilateral width of articular facet for occipital condyles, crest to crest	73.28
height of articular facet for axis	25.74
bilateral width of articular facets for axis, crest to crest	65.92
height of transverse process	26.92
height of spinous process, as preserved	14.93
axis	
height, tip of neural spine to ventral margin of hypapophysis	85.56
transverse width, left transverse process to midline x2	89.86^*b*^
anteroposterior length, ventrally	35.34
height of neural canal, posteriorly	18.35
width of neural canal, posteriorly	27.21
width of posterior epiphysis	34.98
height of posterior epiphysis, including hypapophysis in midline	33.10
width across margins of atlantal facets, left side to approximate midline x2	66.88^*b*^
C3	
height, tip of neural spine to ventral margin of hypapophysis, neural spine possibly missing	58.34
transverse width, left transverse process to midline x2	95.76^*b*^
anteroposterior length, ventrally, including hypapophysis	16.08
height of neural canal, posteriorly	14^*c*^
width of neural canal, posteriorly	23^*c*^
width of posterior epiphysis	34.02
height of posterior epiphysis, including hypapophysis in midline	36.79
maximum dimension of vertebrarterial canal, left	11.49
C4	
height, lacks neural arch	55.25^*c*^
transverse width, right transverse process to midline x2	73.32^*b*^
anteroposterior length, ventrally, including hypapophysis	16.27
anteroposterior length, ventrally, adjacent to hypapophysis	15.31
height of neural canal, taken posteriorly	11.30^*c*^
width of neural canal, taken posteriorly, right pedicle to midline x2	31.46^*b*^
width of posterior epiphysis	33.03
height of posterior epiphysis, including hypapophysis in midline	39.47
maximum dimension of vertebrarterial canal, right	17.35
C5	
height, damaged neural arch	55.88^*c*^
transverse width, left transverse process to midline x2	106.00^*b*^
anteroposterior length, ventrally, including hypapophysis	16.65
anteroposterior length, ventrally, adjacent to hypapophysis	16.14
height of neural canal, arch lost in midline	16.50^*c*^
width of neural canal, posteriorly, left pedicle to midline x2	34.80^*b*^
width of posterior epiphysis	34.19
height of posterior epiphysis	38.85
maximum dimension of vertebrarterial canal	17.31
C6	
height, damaged neural arch	53.60^*c*^
transverse width, left transverse process to midline x2	105.80^*b*^
anteroposterior length, ventrally	17.42
height of neural canal, arch lost in midline	12.63^*c*^
width of neural canal, posteriorly, left pedicle to midline x2	40.64
width of posterior epiphysis	37.44
height of posterior epiphysis	37.89
maximum dimension of vertebrarterial canal	19.44
C7	
height, crushed neural arch	58.00^*c*^
transverse width, left transverse process to midline x2	94.06
anteroposterior length, ventrally	18.89
height of neural canal, crushed neural arch	14.20^*c*^
width of neural canal, posteriorly, crushed neural arch	
width of posterior epiphysis, distorted	37.60
height of posterior epiphysis	34.14^*c*^
T1	
height, incomplete neural spine	58.93
transverse width, left transverse process to midline x2	75.32^*b*^
anteroposterior length, ventrally	20.33
height of neural canal, crushed neural arch	15.56^*c*^
width of neural canal, posteriorly, left neural pedicle broken	30.50
width of anterior epiphysis, posterior damaged	35.34^*a*^
height of anterior epiphysis	33.58
T2	
height, incomplete neural spine	—
transverse width, right transverse process to midline x2	80^*a*^
anteroposterior length, ventrally	23.65
height of neural canal, crushed neural arch	—
width of neural canal, posteriorly, left neural pedicle broken	28.25^*c*^
width of anterior epiphysis, posterior damaged	33.90^*a*^
height of anterior epiphysis	30.33^*a*^
T3	
height, incomplete neural spine	—
transverse width	64.80^*c*^
anteroposterior length, ventrally	25.40
height of neural canal, crushed neural arch	—
width of neural canal, posteriorly, pedicles damaged	29.90^*a*^
width of posterior epiphysis	40^*a*^
height of posterior epiphysis	30.03
T4	
height, incomplete neural spine	57.26^*c*^
transverse width, left transverse process to midline x2	88^*b*^
anteroposterior length, ventrally	27.77
height of neural canal, anterior, posterior crushed	15.02
width of neural canal, crushed, pedicles deformed	24.20
width of posterior epiphysis	40^*a*^
height of posterior epiphysis	30.80^*a*^
T5	
height	114.40
transverse width, left transverse process to midline x2	80.44^*b*^
anteroposterior length, ventrally	28.79
height of neural canal, posterior, anterior deformed	18.40^*a*^
width of neural canal, pedicle damaged	26.60^*a*^
width of posterior epiphysis	41.21^*a*^
height of posterior epiphysis	31.88
T6	
height	121.26
transverse width, left transverse process to midline x2	75.2^*a*^
anteroposterior length, dorsally (ventral eroded)	25.9^*a*^
height of neural canal, anterior, posterior deformed	17.74
width of neural canal, pedicle damaged	27.16
width of anterior epiphysis	32.83
height of anterior epiphysis	30.71

^*a*^Estimated measurement.

^*b*^The measurements are made from one side to the midline of the skull and multiplied by two.

^*c*^Loss of part of a feature, so that the dimension is normally a minimum.

#### 4.2.1 Atlas

**Figure 13.**
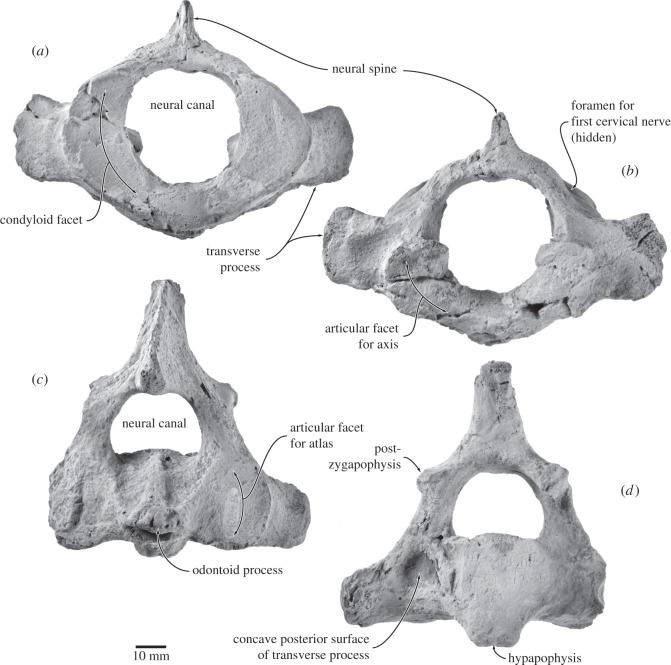
Atlas and axis of *Fucaia buelli*. Axis in (*a*) anterior and (*b*) posterior view; atlas in (*c*) anterior and (*d*) posterior view.

In anterior view, the atlas (UWBM 84024A) is dominated by a large, circular neural canal (figure 13*a*). The concave condyloid facets are crescentic and separated from each other ventrally. Ventrally, the atlas bears an indistinct, rounded surface possibly representing an incipient hypapophysis. The single transverse process is robust, imperforate and oriented slightly dorsolaterally. The neural arch barely rises above the level of the dorsal edge of the articular facets. In posterior view, the tabular articular facets for the axis are damaged, but seem to have been reniform and located somewhat ventral to the level of the transverse processes (figure 13*b*). An oblique ridge descends forwards from the dorsal margin of each facet and separates the fovea dentis from the neural canal *sensu* *stricto*; the transverse ligament probably arose from this ridge.

In lateral view, the atlas is by far the most robust of all the cervical vertebrae. The condyloid facets and the facets for the axis are not parallel, but oriented at an angle of *ca* 18°. Ventral to the spinous process and anterodorsal to the transverse process, the neural arch carries the foramen for the first cervical nerve and vertebral artery (figure 13*b*). The transverse process is anteroposteriorly compressed and twisted relative to the main vertical axis of the vertebra (clockwise on the left, anticlockwise on the right). The spinous process is short and oriented dorsally, with its apex being somewhat eroded; posterodorsally, the process is excavated to accommodate the axis. In dorsal view, the anterior margin of the neural arch is broadly concave, whereas the posterior margin is comparatively straight.

#### 4.2.2 Axis

In anterior view, the general profile of the axis (UWBM 84024B) is triangular, comprising a transversely broad body bearing the articular facets for the atlas, a dorsally narrowing neural canal roughly equalling the body in height, and a tall spinous process (figure 13*c*). The odontoid process is well developed and bears a prominent median ridge on its dorsal surface that extends posteriorly to the posterior border of the body. Anterolaterally, this ridge is flanked by a transverse depression with a sharply defined lateral border, clearly separating it from the articular facet for the atlas. The transverse process is short transversely but tall dorsoventrally, similar in height to the articular surface for the atlas. There is no transverse foramen/vertebrarterial canal.

In lateral view, the odontoid process is conical. The transverse process is oriented somewhat posterolaterally and resembles that of the atlas in being anteroposteriorly compressed and twisted. The neural arch is robust and bears well-developed postzygapophyses. The spinous process is elongate anteroposteriorly and roughly triangular, extending anteriorly far beyond the pedicle of the neural arch and the odontoid process to articulate with the spinous process of the atlas. In posterior view, the body is oval to sub-circular in outline and confluent with a blunt, bifid, robust, posteroventrally directed hypapophysis (figure 13*d*). Immediately lateral to the body the posterior surface of the transverse process is distinctly concave. The articular facet of the postzygapophysis is oriented ventrolaterally and located just ventral to the level of the spinous process.

#### 4.2.3 C3–C7

**Figure 14.**
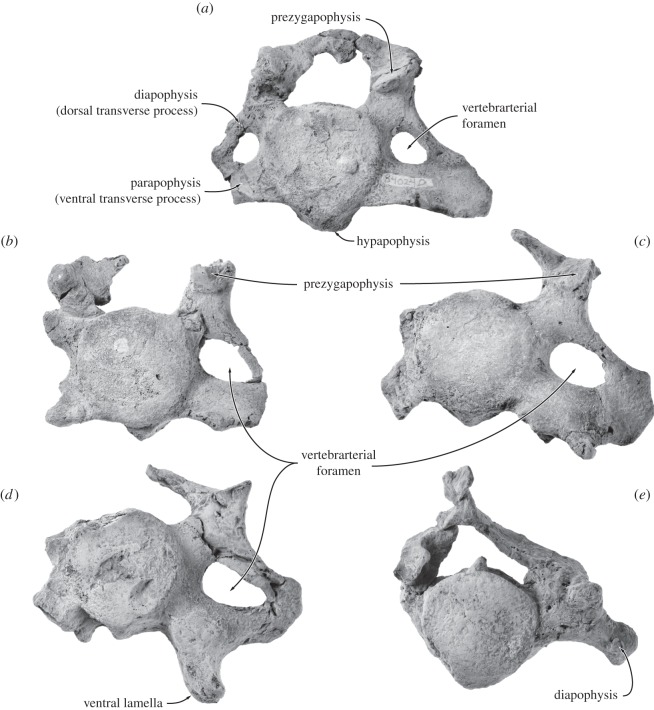
Posterior cervical vertebrae of *Fucaia buelli*. (*a*) C3, (*b*) C4, (*c*) C5, (*d*) C6, (*e*) C7. All in anterior view.

The posterior cervical vertebrae (UWBM 84024C–G, with C7 labelled out of sequence as UWBM 84024C) resemble each other in general shape and size (figure 14). All show some distortion and/or loss, hampering consistent measurements (table 4). Anterior and posterior epiphyses are fused; height and width are estimates of where the epiphysis merges with the body. The body is sub-circular in anterior view, compressed anteroposteriorly and ventrally terminates in a hypapophysis that is prominent and slightly bifid on C3 but thereafter reduces to a ridge. The relative size of the hypapophysis is consistent with a large ventral longitudinal ligament anteriorly (especially on the axis and C3), reducing posteriorly to a median ventral crest in C5–C6, and to a lower wider crest in C7 in which the profile of the anterior face is slightly cordiform.

C3–C6 bear distinct dorsal (diapophyses) and ventral (parapophyses) transverse processes enclosing an increasingly larger vertebrarterial foramen, with the parapophysis being significantly more robust than the gracile diapophysis. In C5 and especially C6 the parapophysis is extremely developed, with its dorsolateral corner pointing posterodorsally to meet the diapophysis and its ventrolateral corner giving rise to a well-developed ventral lamella (figure 14*c*,*d*). In C7, the parapophysis is absent. The prezygapophysis, which on C3 is located dorsal to the lateral edge of the body, gradually shifts further laterally and ventrally on successive vertebrae. In lateral view, both the neural arch and the prezygapophysis become increasingly elongated, with the latter projecting well beyond the anterior face of the body. The spinous process is largely missing in C3–C6; in C7 it is damaged, but seems to have been short and bluntly rounded. Posterolaterally, the diapophysis of C7 bears what appears to be a costal facet. If so, this would imply the presence of a supernumerary cervical rib, which occasional also occurs in extant mysticetes [32]. C3 and C4 have slightly concave epiphyses; the anterior epiphyses become more convex from C5 to C7, while the posterior epiphyses are progressively concave.

#### 4.2.4 Thoracic vertebrae

**Figure 15.**
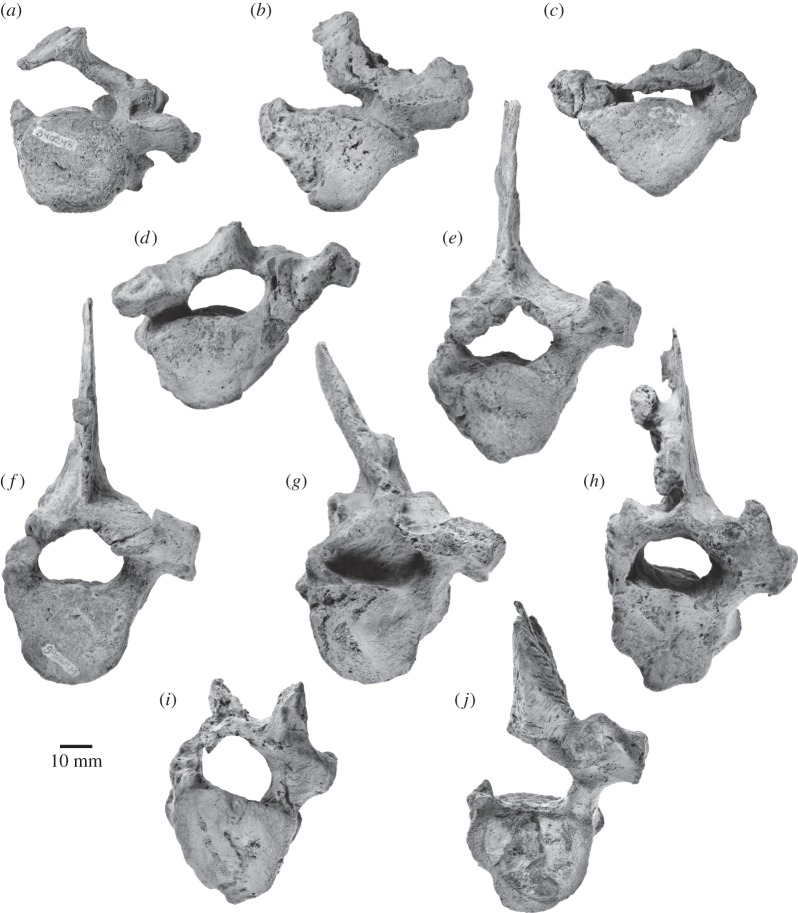
Thoracic vertebrae of *Fucaia buelli*. (*a*–*e*) T1–T5, (*f*) T6, (*g*–*j*) posterior thoracic vertebrae of uncertain identity. All in anterior view.

Ten variously damaged and deformed thoracic vertebrae increase markedly in size posteriorly (figure 15; table 4). The nature of preservation precludes consistent measurements. T1–T5 (UWBM 84024H–L) can be identified with confidence. UWBM 84024M may represent T6, but the loss of much of its body prevents clear identification. Judging from the length of their bodies, the final four vertebrae (UWBM 84024N–P, plus one without letter suffix) belong to the posterior part of the sequence, but it is impossible to tell how many, if any, additional thoracics may be missing. In anterior view, the outline of the vertebral body is initially ovoid to cordiform, but becomes increasingly circular from T5 onwards. T1–T4 have a bifid or nodular posterior hypapophysis. The transverse process is robustly built and transversely short throughout the entire series. The spinous process is missing in T1–T4; where preserved, it is transversely compressed and about as high dorsoventrally as the body and neural canal combined. In lateral view, both the body and the neural arch gradually increase in anteroposterior length. A recognizable metapophysis first occurs in T5, and becomes progressively more pronounced and located closer to the spinous process in more-posterior vertebrae. The transverse (tubercular) costal facet is ovoid and oriented anterodorsally on T1–T5 and UWBM 84024M, but circular on all of the more-posterior thoracics. A semi-facet for articulation with the head of the corresponding rib is present on all of the vertebral centra. Where preserved, the spinous process is broad anteroposteriorly and oriented almost vertically.

#### 4.2.5 Lumbar vertebrae

**Figure 16.**
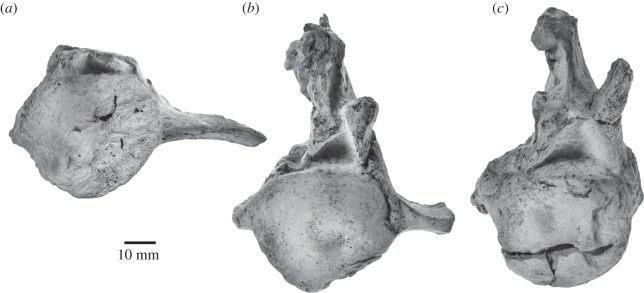
Lumbar vertebrae of *Fucaia buelli*. All in anterior view.

The three lumbar vertebrae (figure 16) preserved with the specimen are characterized by anteroposteriorly elongated centra with an ovoid anterior outline and a ventral carina. The spinous processes are mostly lost, but seem to have been robust and oriented posterodorsally in lateral view. The transverse processes are broken, although their approximate shape can be surmised from their dorsoventrally compressed and anteroposteriorly elongate bases. One of the vertebrae preserves a well-developed, dorsally pointing metapophysis.

#### 4.2.6 Ribs

Numerous rib fragments (not figured) provide little information regarding their original number, position and morphology. Two of the fragments are transversely broadened and thus may represent the left and right first rib, respectively.

#### 4.2.7 Forelimb

**Figure 17.**
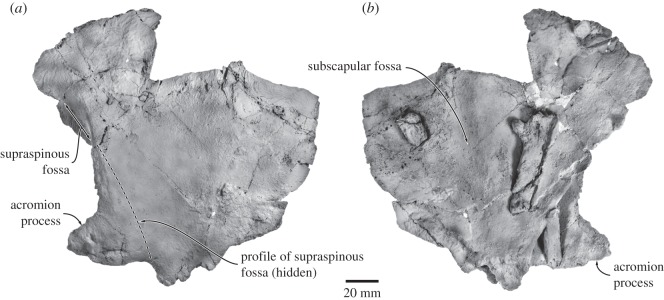
Forelimb of *Fucaia buelli*. Left scapula in (*a*) lateral and (*b*) medial view.

The forelimbs are almost completely lost, except for the fragmentary left scapula and a heavily eroded radius (figure 17). The shape of the scapula is uncertain owing to breakage around the margins. Judging from its robust base, the acromion process was probably well developed. The supraspinous fossa is relatively narrow and confined to the anteriormost portion of the scapula, yet deep and delineated by a tall scapular spine. Towards the dorsal rim of the scapula, the spine weakens and, ultimately, disappears. The infraspinous fossa is broad, shallow and largely featureless, except for a low dorsoventral ridge running through its centre.

## 5. Discussion

### 5.1 Phylogeny

**Figure 18.**
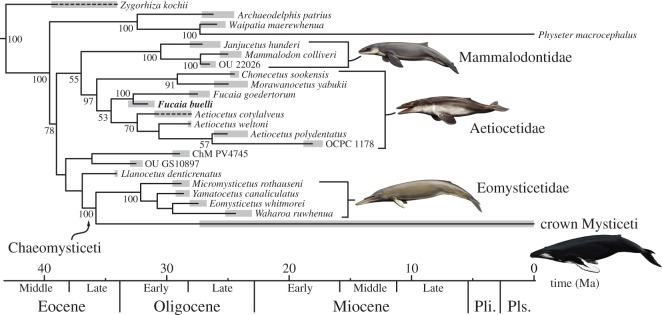
Phylogenetic relationships of archaic mysticetes. Individual families are labelled. For details of the analysis, see Marx and Fordyce [13]. Abbreviations: Pli, Pliocene, Pls., Pleistocene.

After 50 million generations, our Bayesian total evidence analysis showed a reasonable level of convergence, with an average level of split frequencies less than 0.015. The resulting consensus tree resembles that of Marx & Fordyce [13] in placing *F. buelli* as sister to *F. goedertorum* and, along with the latter, within a monophyletic Aetiocetidae (figure 18; electronic supplementary material, figure S1). Aetiocetids in turn cluster with mammalodontids in a ‘toothed mysticete’ clade that is basal to a variety of other toothed taxa and chaeomysticetes. However, our results differ from those of Marx & Fordyce [13] in two important respects: (i) OCPC 1178, an as yet undescribed Early Miocene specimen from California, is now located inside *Aetiocetus* as sister to *A. polydentatus*; little can as yet be said about this result, except that it is unsurprising in light of the young age of both OCPC 1178 and *A. polydentatus* relative to other members of *Aetiocetus*; and (ii) *Fucaia* is now sister to *Aetiocetus*+OCPC 1178, to the exclusion of *Morawanocetus*and *Chonecetus*.

The equally weighted parsimony analysis yielded 4770 most parsimonious trees (MPTs) of 1216 steps each (CI=0.296, RI=0.734). Despite this large number of MPTs, the strict consensus is relatively well resolved (electronic supplementary material, figure S2) and overall similar to the results of Marx & Fordyce [13]. *Fucaia buelli* is again sister to *F. goedertorum* (with a reasonable branch support of 70%) and clusters with *Aetiocetus* and OCPC 1178 to the exclusion of *Chonecetus* and *Morawanocetus*; however, OCPC 1178 is now outside of *Aetiocetus*, and the interrelationships of Mammalodontidae, Aetiocetidae, Chaeomysticeti and a range of archaic toothed mysticetes (including *Llanocetus*) are unresolved.

By contrast, implied weighting (3 MPTs, fit=165.53, CI=0.290, RI=0.725) resolves basal mysticete relationships in mostly the same way as the Bayesian analysis, with mammalodontids and aetiocetids forming a clade basal to *Llanocetus*+Chaeomysticeti (electronic supplementary material, figure S3). As before, *F. buelli* and *F. goedertorum* are closely related and group with *Aetiocetus* to the exclusion of *Morawanocetus*. There are, however, also some marked differences. Thus, implied weighting causes *Ch. sookensis* to cluster with *Fucaia*, *contra* the results of both the Bayesian and the equally weighted parsimony analyses. In addition, OCPC 1178 is still located outside *Aetiocetus*, and the poorly known ChM PV474 now clusters with Aetiocetidae+Mammalodontidae instead of chaeomysticetes. On the whole, the Bayesian and parsimony analyses agree in identifying *F. buelli* as an aetiocetid, in confirming the monophyly of both *Fucaia* and Aetiocetidae, and in placing *Morawanocetus* at the base of the family as a whole. The position of *Ch. sookensis* and other, non-aetiocetid toothed mysticetes is more ambiguous. Nevertheless, at least two of our analyses corroborate each other in placing *Chonecetus*outside the clade formed by *Aetiocetus*+*Fucaia*, and in replicating the basal mysticete branching pattern proposed by Marx & Fordyce [13], respectively.

In the following, we will primarily discuss the outcome of the Bayesian total evidence analysis. Our results concur with those of most previous studies in finding aetiocetids to be monophyletic [5,6,8,33,34], although some recent analyses have questioned this idea [9,26]. The features that place *F*. *buelli* most clearly into Aetiocetidae are (i) the presence of an embayment for the lacrimal bone in the lateral border of the ascending process of the maxilla; and (ii) the presence of strong enamel ornament on the lingual side of the (cheek) teeth only. Other characters potentially diagnostic of this group, such as a centrally constricted mandible, the presence of a notch in the posterior margin of the palatine and a finger-like coronoid process of the mandible, are not preserved in *F. buelli*, although they are predicted to have been present judging from its phylogenetic position and overall resemblance to *F. goedertorum*.

**Figure 19.**
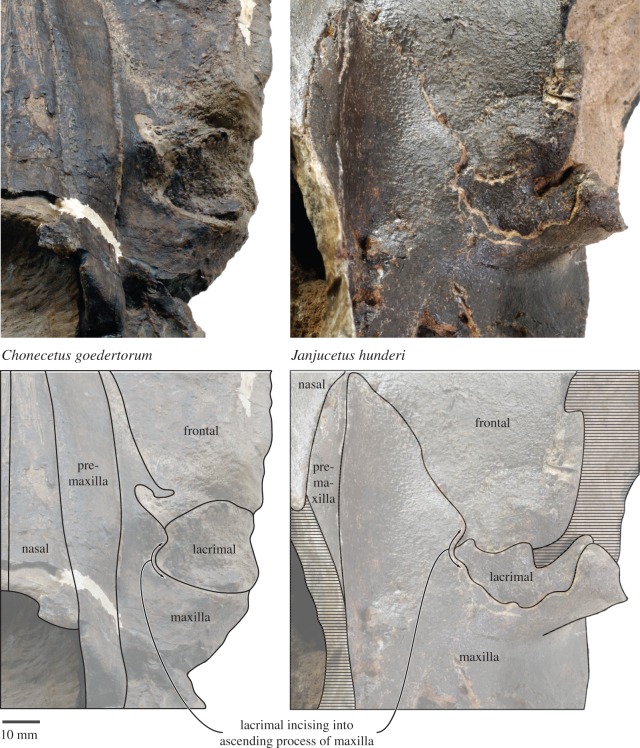
Position of the lacrimal relative to the ascending process of the maxilla in *F. goedertorum* and *Janjucetus hunderi*. *C. goedertorum* (left) is represented by LACM 131146, *J. hunderi* (right) by NMV P216929.

The presence of an (enlarged) lacrimal incising into the ascending process of the maxilla was previously interpreted by Deméré & Berta [6] as an unequivocal synapomorphy of a clade comprising *Fucaia* (=*Chonecetus* of Deméré and Berta)+*Aetiocetus*. Such a lacrimal structure may be diagnostic, but cannot currently be demonstrated because the lacrimal is unknown in *Ch. sookensis* and *Morawanocetus*. It is possible that a lacrimal embayment in the ascending process of the maxilla is characteristic of a larger clade, such as Aetiocetidae as a whole or even Aetiocetidae+Mammalodontidae. Notably, *Janjucetus* also has a sizeable lacrimal bone that slightly incises into the adjacent ascending process of the maxilla (figure 19). By contrast, this feature appears to be absent in *Mammalodon*, although the loss of the lacrimals and the dislodgement and partial distortion of the ascending processes of the maxillae make it difficult to reconstruct this portion of the skull.

The second character—the presence of ornamented enamel on the lingual side of the teeth only—also requires comment. Deméré & Berta [6] interpreted this feature as a synapomorphy of the genus *Aetiocetus* only and coded it as ‘absent’ in *Morawanocetus*, which instead was described as having enamel ridges on both sides of the teeth. By contrast, Marx & Fordyce [13] coded one-sided enamel ornamentation as ‘present’ in *Morawanocetus*. Additional observations reveal this as a coding error, and we now follow Deméré & Berta [6] in regarding *Morawanocetus yabukii* as having distinct vertical enamel ridges on both sides of the premolar and molar tooth crowns. Nevertheless, *F. buelli*demonstrates that this character state is not restricted to *Aetiocetus*, and thus is probably diagnostic of a more inclusive clade also including *Fucaia*.

Until now, *F. goedertorum* was included in *Chonecetus*. The latter in turn is based on *Ch. sookensis*, which is currently only known from its poorly preserved holotype [1,35]. Previous phylogenetic analyses reconstructed *Ch. sookensis* as either sister to *F. goedertorum*(albeit with low branch support [8]) or *M. yabukii* [13]. In our present analysis, *Ch. sookensis* and *M. yabukii* are grouped mainly by characters relating to the supraoccipital, including its flattened dorsal surface and, possibly, the absence of an external occipital crest. Together, these two species represent the earliest diverging aetiocetids, as previously suggested for *Morawanocetus* only [6]. Nevertheless, this topology needs to be interpreted with some caution: *Fucaia* and *Aetiocetus* clearly share distinctive features not seen in *Morawanocetus*, including lingual-only enamel ornament, fused or closely apposed postcanine roots, a posteriormost tooth situated in front of (rather than below) the orbit, the absence of a shelf-like entocingulum and the presence of a central constriction in the body of the mandible (see also [36], fig. 3); yet none of these characters are known in *Ch. sookensis*, which means that the actual position of *Chonecetus* remains uncertain for now.

Our analysis differs from most other published studies in recovering aetiocetids as basal relative to other toothed mysticetes. A striking example of this is ChM PV4745, which is usually placed at the base of the mysticete tree (e.g. [8,26,27]), but allied with chaeomysticetes, to the exclusion of aetiocetids, in our analysis. The position of *L. denticrenatus* is also surprising, given the latest Eocene age and archaic dental morphology of this taxon [10,12]; however, previous analyses have been less consistent in identifying its affinities, clustering it either with mammalodontids or, as found here, chaeomysticetes [8,34]. These discrepancies at least partly may stem from the current lack of detailed morphological descriptions of both *Llanocetus* and ChM PV4745, as well as the non-preservation of critical character states, such as the condition of the mandibular symphysis. We therefore expect that future analyses may place these taxa more basally than our current results, but emphasize that, as long as aetiocetid monophyly remains intact, such changes would not affect our proposals regarding the evolution of mysticete feeding strategies detailed below.

### 5.2 Body size

All of the cervical and probably all of the thoracic vertebral epiphyses appear to be fused, which suggests that UWBM 84024 is an adult [37]. This estimate is consistent with the presence of erupted molars showing attritional and abrasive wear, but is unexpected given the presence of the partly unfused supra-exoccipital suture, which in extant mysticetes completely ossifies before the age of one [38]. Fontanelles and/or breaks coinciding with the supra-exoccipital suture are not uncommon among aetiocetids and, besides *F. buelli*, also occur in *F. goedertorum*(LACM 131146), *Ch. sookensis* (CMN FV 12095) and *M. yabukii* (AMP 01). The frequent occurrence of this juvenile trait may reflect paedomorphism, as previously suggested for both aetiocetids and mammalodontids [8,39].

Breakage prevented us from taking accurate measurements, but, based on what has been preserved, we estimate bizygomatic width to have been about 210 mm. Inserted into the regression equations provided by Pyenson & Sponberg [40, supplementary eqn (vi)] and Lambert *et al*. [41, supplementary figure 9], this results in a total length estimate for *F. buelli* of approximately 1.7–1.8 m. These figures seem plausible, but need to be interpreted with caution: in the study by Pyenson & Sponberg [40], computations based on bizygomatic width underestimated the length of the kentriodontid *Atocetus* by 18%, but overestimated that of the balaenopterid *Balaenoptera siberi* by 47%. This discrepancy may be related to taxon-specific constraints, such as particular functional demands placed on *B. siberi* by its lunge-feeding strategy, or reflect differences in allometric scaling. In terms of both its size and likely feeding ecology, *F. buelli* arguably resembles *Atocetus* more than *B. siberi*, and hence we suspect that our initial estimate of 1.7–1.8 m may be too small. Assuming the latter to underestimate real length by 18%, as in *Atocetus*, a total length of 2.1–2.2 m may be more realistic, but cannot currently be demonstrated for lack of direct evidence.

Judging from its bizygomatic width [41], *F. goedertorum* was approximately the same size as *F. buelli*. The body size of *Fucaia* thus is markedly below estimates for virtually all other described mysticetes, including mammalodontids such as *Janjucetus hunderi* (2.9–3.2 m) and other aetiocetids such as *A. cotylalveus* (2.8–3.0 m) [40]. *Ch*. *sookensis* is the only mysticete that may have been slightly smaller (*ca* 1.6 m, or 1.9–2.0 m if the initial figure is assumed to be an underestimate); however, this size difference between *Fucaia* and *Chonecetus* is exaggerated by the different ontogenetic ages of the available material. Thus, fusion of the vertebral and humeral epiphyses suggests that the holotype of *F. goedertorum* (LACM 131146), like that of *F. buelli*, is an adult or, possibly, subadult. By contrast, the holotype of *Ch. sookensis* (CMN FV 12095) includes a caudal vertebra that seems to lack the posterior epiphysis, and hence is more likely a juvenile. Remarkably, *Fucaia* and *Chonecetus* seem to exceed the body length of the smallest known cetacean, the vaquita (*Phocoena sinus*), by less than 1 m [42]. This not only highlights the diminutive nature of many of the earliest baleen whales, but defines the lower bound of an extraordinarily broad size range in aetiocetids in particular, and living and extinct mysticetes in general [40,43].

### 5.3 Feeding strategy

#### 5.3.1 Raptorial feeding

Until now, the feeding strategy of *Fucaia* has remained unclear owing to the loss of virtually all teeth in both available specimens of *F. goedertorum*. *F. buelli* now fills this gap, and demonstrates that, like *Morawanocetus*, *Fucaia* has a markedly heterodont dentition capable of both prehension and, presumably, mastication (shearing) of prey items in the manner proposed for archaeocetes [44,45]. Wear facets on the posterior lingual faces of the upper cheek teeth imply occlusion by the anterior labial faces of the opposing mandibular teeth. The occlusion and wear are consistent with diastemata being small, as in *F. goedertorum*, and suggest that *Fucaia* was mainly an archaeocete-like raptorial feeder. In size and complexity, the teeth in *Fucaia* contrast with those of members of *Aetiocetus*, all of which have more gracile, widely spaced tooth crowns that probably were incapable of efficient mastication—even though they, too, show distinctive wear facets suggesting function in prey capture [1,3,6].

#### 5.3.2 Transitional morphology and suction feeding

Aetiocetids have been said to have an intermediate morphology of the feeding apparatus that combines a functional dentition with some form of incipient baleen [5]. In extant mysticetes, the baleen racks are nourished by branches of the superior alveolar artery, which in turn are carried by a series of large, easily recognizable palatal nutrient foramina and sulci occupying much of the ventral surface of the maxilla [46]. Similar, albeit much smaller, foramina and sulci also occur in *Aetiocetus*, *Fucaia* and *Morawanocetus* [5,36], although their presence and/or homology have been questioned [8,36,47,48]. If the palatal foramina of aetiocetids are indeed homologous with those of modern mysticetes, why are such foramina absent in the putative sister taxon of Aetiocetidae (figure 18), the mammalodontids [8,9]? There are two possible answers: (i) the results of our analysis are wrong and aetiocetids are instead more closely related to chaeomysticetes (e.g. [5,8]); or (ii) the absence of palatal nutrient foramina and sulci in mammalodontids implies either a loss or a dual origin of baleen [33,46].

Given the highly specialized nature of baleen, we consider it highly unlikely that it originated twice within Mysticeti, but secondary loss in mammalodontids is conceivable. Consider two observations: first, as noted above, both aetiocetids and mammalodontids have been interpreted as potentially paedomorphic [8,39]. There is no quantitative evidence backing up this idea, but the frequent occurrence of a partly unfused supra-exoccipital suture and/or fontanelles among aetiocetids, as well as the small body size, large orbits and relatively large occipital condyles characterizing both taxa are suggestive. Furthermore, heterochrony would provide a relatively straightforward explanation for the absence of palatal nutrient foramina in mammalodontids, considering that even extant mysticetes first go through a developmental (fetal) stage in which they possess *only* teeth [5,49].

Second, as noted by Ichishima [50], even if aetiocetids had some form of baleen precursor, it does not necessarily follow that the latter was employed in filter feeding. Indeed, teeth used in raptorial feeding might have interfered with, rather than complemented, a separate filtering apparatus consisting of individual baleen plates. This is especially true for taxa with relatively large teeth, such as *M. yabukii* and *F. buelli*. In these, any baleen between the teeth could have prevented effective shearing, while mastication in turn could have left the baleen rack in disarray.

Instead of baleen, some aetiocetids may have possessed heightened gums, as has been suggested for mammalodontids based on their strongly emergent tooth crowns [8]. Enlarged gum tissue might have been better able to accommodate bites, allowing a mostly raptorial feeding strategy, while having had the added effect of at least partially sealing the lateral gape when the mouth was nearly closed. This seal, in turn, could have assisted in generating suction to transport partially dismembered and or/small prey—initially captured with the anterior teeth—towards the back of the mouth for swallowing, as seen in many extant odontocetes [51,52]. Note, however, that the teeth in *F. buelli* show no particular evidence for or against heightened gums.

Perhaps against the idea of any significant suction capabilities in aetiocetids stands the relatively gracile nature of the hyoid apparatus, which in extant suction feeding odontocetes is often conspicuously enlarged [53]. The relative size of odontocete hyoid bones is, however, variable even among specialized suction feeders [31] and only partially reflective of suction capability [54]. In addition, even largely raptorial taxa with a comparatively small hyoid apparatus might use suction to transport or even acquire prey [55,56]. Maybe the most important muscle involved in suction feeding is the sternohyoideus, which originates from the manubrium of the sternum and pulls the basi- and thyrohyals, and thus ultimately the tongue, backwards, resulting in the generation of negative intraoral pressure [55]. Fitzgerald [8] used the presence of an enlarged manubrium (among other features) in *Mammalodon colliveri* to argue for suction feeding, even though the hyoid apparatus of this species is comparatively small. A massive manubrium also occurs in aetiocetids (including *Fucaia*) and even basal chaeomysticetes [57], which implies that both it and a well-developed sternohyoideus muscle may be typical of archaic mysticetes in general.

#### 5.3.3 Unsutured mandibular symphysis and dental simplification

Besides palatal nutrient foramina, aetiocetids share with modern mysticetes the presence of an unsutured mandibular symphysis allowing longitudinal (alpha) rotation of the lower jaws and—at least in *Aetiocetus*—incipient homodonty and polydonty [1,5,58]. Extant rorquals and balaenids employ mandibular rotation to expand the mouth cavity and move the lower lips during intermittent and continuous ram feeding, respectively [59,60]. In *Fucaia*, however, tooth wear patterns indicate exact occlusion, and thus little or no alpha rotation; the mandibular symphysis, though unsutured, was probably firmly ligamentous. Similarly, alpha rotation may have been absent in other aetiocetids, judging from the presence of wear facets in *Aetiocetus* and the observation that the tall coronoid process was probably constrained within the temporal fossa [6,61]. Nevertheless, it is possible that at least some aetiocetids might have used minor rotation of the mandibles to facilitate (benthic?) suction feeding via enhanced lip control, as seen in extant *Eschrichtius* and, maybe, cetotheriids [62–64], or to avoid trapping the enlarged gums and/or proto-baleen between the jaws during mouth closure [65].

**Figure 20.**
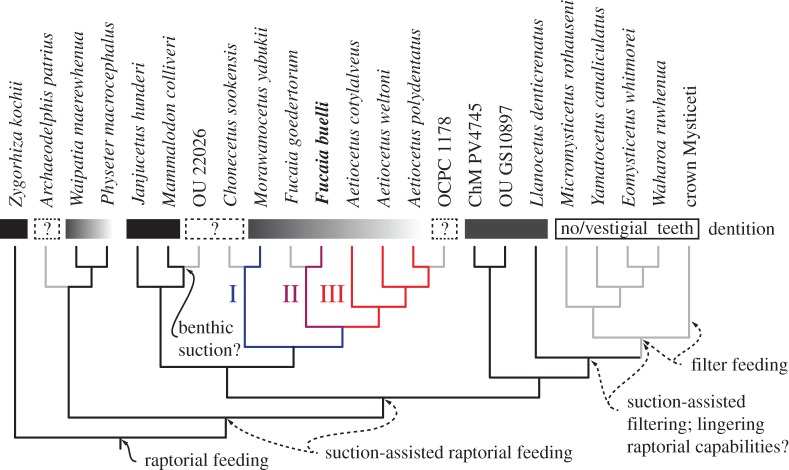
Proposed evolution of mysticete feeding strategies. Phylogeny is the same as in figure 18. Boxes above the branches indicate the type of dentition, with black denoting heterodonty, white homodonty and grey the transition between. Roman numerals refer to the three stages of aetiocetid tooth simplification explained in the text. Stippled lines indicate uncertainty about when a particular feeding strategy first appeared.

The dental morphology of *Fucaia* forms part of a morphological transformation series leading from the robust, closely spaced and denticulate teeth of basilosaurid archaeocetes to the slender and nearly homodont teeth of *Aetiocetus*. This transition can roughly be divided into three stages (figure 20). First, as in *Morawanocetus*, aetiocetid tooth crowns acquired prominent labial and lingual enamel ornament (shared with mammalodontids and llanocetids), but the crowns also became lower, more widely spaced, and lost the originally shelf-like ectocingulum. Second, as in *Fucaia*, the entocingulum was also reduced and the crowns became more gracile and transversely compressed, while remaining effective for shearing. Finally, as in *Aetiocetus*, the crowns became separated from each other by large diastemata, conical and less obviously denticulate. This final stage is also the first to show polydonty, with 12 teeth present in the mandible of *A. weltoni* and 14–15 in that of *A. polydentatus*. Note that fetuses of extant baleen whales also possess a polydont and seemingly homodont dentition [5,49]; however, they probably acquired both of these traits independently, given the archaic dentitions of *Fucaia* and *Morawanocetus* and the prevailing view of aetiocetid monophyly [5,6,8,13,33].

The trend towards a simplified, expanded dentition is reminiscent of the situation in many extant odontocetes, in which the teeth function mainly in capturing food, rather than mastication, with ingested items being sucked directly to the rear of the oral cavity [51,66]—as suggested above for aetiocetids. A similar scenario could explain how incipient filter feeders might still use teeth to capture large individual prey [5]. However, we question how the coexistence of masticating, closely spaced teeth and proto-baleen might have worked in practice. Interestingly, tooth structures related to raptorial and filter feeding are clearly separated in one of the only extant marine mammals known to switch between both strategies: in the leopard seal *Hydrurga leptonyx*, the incisors and canines pierce large prey, whereas the postcanine teeth are mostly used for sieving [67]. Similarly, a separation of the posteriorly located filtering portion of the feeding apparatus and the area of the rostrum retaining teeth has been proposed for the eomysticetid *Waharoa ruwhenua* [68]. This is unlike the situation in *Aetiocetus*, in which polydonty, incipient homodonty, and diastemata are expressed along the entire tooth row, and thus would have overlapped with the baleen racks.

#### 5.3.4 Transition to filter feeding

Whether aetiocetids would initially have used suction merely to transport prey to the back of the mouth, as opposed to actually capturing it, is uncertain, given that both raptorial feeding followed by (or coexisting with) suction and vice versa have been observed in extant odontocetes and pinnipeds [66,67,69]. In either case, it seems reasonable to propose that purely raptorial feeding—probably still important in *Morawanocetus*and *Fucaia*—waned while that of suction-assisted feeding accordingly waxed. In aetiocetids, teeth remained essential for prey capture and/or processing, especially in clearly heterodont taxa such as *Fucaia*. However, there are archaic mysticetes other than aetiocetids that also might have used suction in feeding.

Mammalodontids, and in particular *Mammalodon*, have also been interpreted as at least facultative suction feeders [8], which raises the possibility that some form of suction—probably in conjunction with raptorial feeding—is ancestral not only for toothed mysticetes but possibly all baleen whales [61]. Indeed, if the palatal foramina and sulci in aetiocetids are (i) homologous with the palatal nutrient foramina and sulci in extant mysticetes and (ii) correlated with enlarged gum tissues and thus maybe suction, then the ancestors of chaeomysticetes might initially have had a similar feeding apparatus. In chaeomysticetes, however, the feeding apparatus became more elaborate: along with increased suction capability, they became able to ingest small prey items that could not be captured with teeth alone, and concurrently evolved an efficient way to expel water.

In filter-feeding pinnipeds, such as leopard and crabeater (*Lobodon carcinophaga*) seals, prey is trapped by, and water expelled via, morphologically complex cheek teeth [67]. A similar function has been suggested for the multi-cusped postcanine teeth of archaic mysticetes, although trapping and water expulsion might not have worked for the simple teeth of *Aetiocetus*, or for teeth that show pronounced occlusal wear (e.g. in *Mammalodon*) and/or are separated by large diastemata (e.g. in *L. denticrenatus*); such dentitions probably did not filter [8,9,50]. Further enlargement and differentiation of the gingiva in the ancestors of chaeomysticetes would have provided an alternative way to accommodate the side effects of suction feeding, and thus may have paved the way for their transformation into baleen and the emergence of genuine filter feeders [47]. Archaic chaeomysticetes such as *W. ruwhenua* may provide an insight into how this transition occurred: as the importance of raptorial feeding declined, teeth were lost first from the posterior portion of the rostrum [68]. Freed from the interference of a working dentition, the posterior gingiva could have become more pronounced and given rise to an incipient form of baleen, while the teeth near the front of the rostrum could initially still have functioned in prey capture. Thus, for a time, tooth-assisted prehension, suction and filter feeding could indeed have complemented each other. Eventually, the anterior teeth would also have lost their function and raptorial feeding would completely have given way to (suction-assisted) filtering and, possibly, skim feeding, as recently suggested by Boessenecker & Fordyce [68].

Besides further work focusing on functional morphology and dental microwear, the ideas presented here could be tested by comparing the stable carbon isotope (*δ*^13^C) composition of aetiocetid teeth with that of chaeomysticetes and odontocetes. Carbon isotope values partially reflect the trophic level at which an aquatic consumer feeds, and thus ought to be lower in filter feeders than in raptorial or suction feeding taxa [70]. In line with this pattern, *Mammalodon* has previously been shown to have relatively high *δ*^13^C values reminiscent of those of odontocetes, which supports its interpretation as a suction feeder [71]. What isotopic value should be expected for aetiocetids is, however, uncertain, as their unique feeding morphology (e.g. small size, relatively delicate teeth and slender jaws) suggests that their prey may have differed from, and maybe was smaller than, that of other toothed mysticetes [8].

## 6. Conclusion

*Fucaia buelli* is a previously unrecognized aetiocetid that extends the range of this family to the Early Oligocene. Along with its congeners, *F. buelli* is among the smallest of known mysticetes, with a size comparable with that of small odontocetes. The heterodont dentition of *F. buelli* probably functioned in prey capture and mastication, which could have compromised the putative ability of aetiocetids to filter feed. Instead, *F. buelli* might have employed a form of raptorial and suction feeding, with suction being used either to capture prey items or to transport them to the back of the mouth following ingestion. Under this scenario, structures homologous with the palatal nutrient foramina and sulci of extant mysticetes would have carried blood vessels nourishing enlarged gingiva, rather than baleen plates as such. We argue that a transition from raptorial feeding, to combined raptorial/suction feeding, to combined suction/filter feeding and, ultimately, to filter feeding is functionally more plausible than a direct switch from a raptorial to a filter feeding strategy.

## Supplementary Material

Figures S1-S3: full results of the Bayesian and parsimony analyses

## Data accessibility

The new morphological codings and the full matrix are available from MorphoBank, project 2238 (full matrix stored in the ‘Documents’ section). This published work and the nomenclatural acts it contains have been registered in ZooBank. The LSID for this publication is: urn:lsid:zoobank.org:pub:D46018F8-8E1D-49A9-9EAA- 7BA73ABE6818.
