# Supplementary material for: A new Early Oligocene toothed ‘baleen’ whale (Mysticeti: Aetiocetidae) from western North America: one of the oldest and the smallest
Source: R Soc Open Sci. 2015 Dec 2;2(12):150476. doi: 10.1098/rsos.150476 (PMC4807455; doi:10.1098/rsos.150476)
Supplement: Figures S1-S3: full results of the Bayesian and parsimony analyses [file rsos150476supp1.pdf]

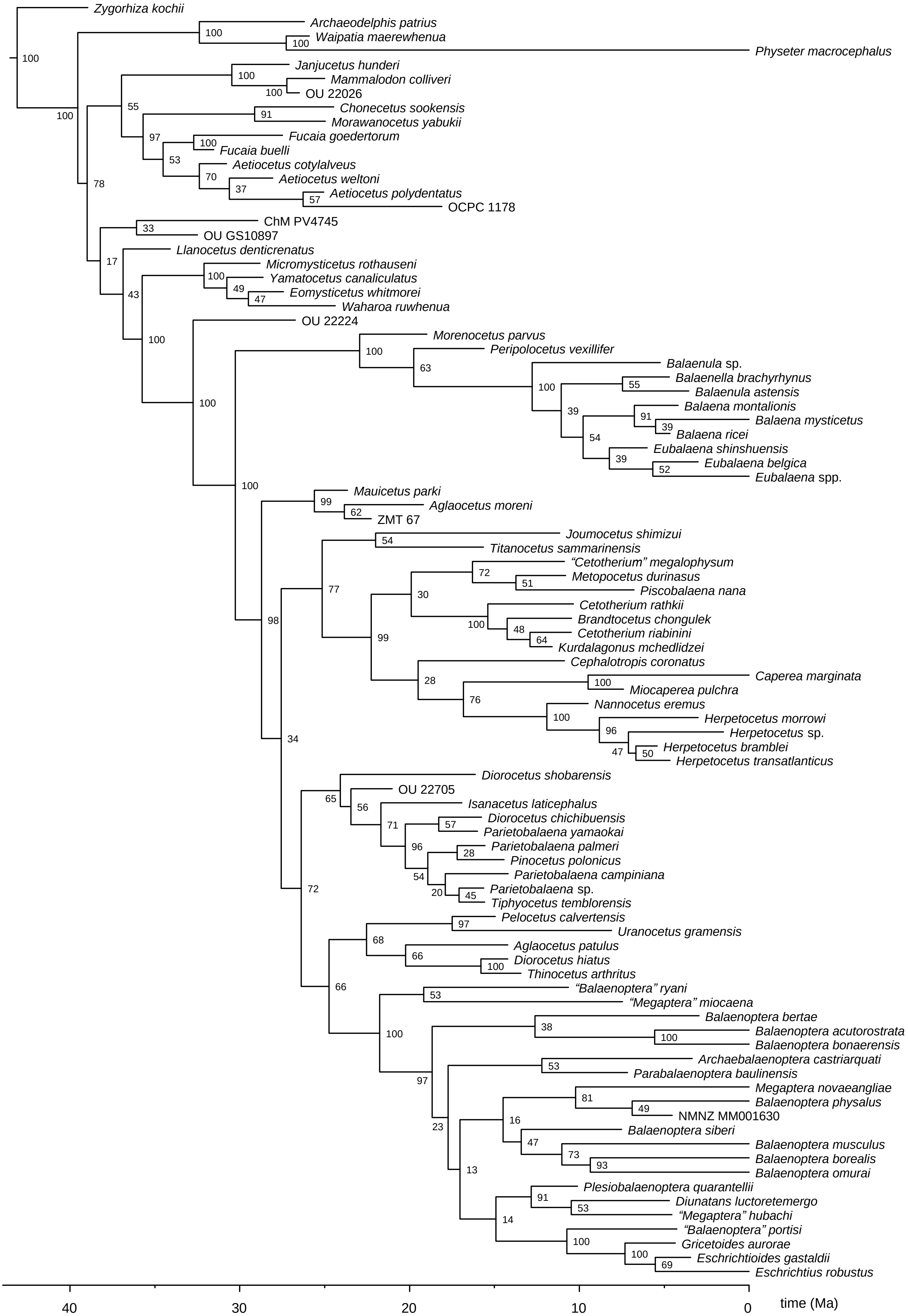

Supplementary Figure S1. Bayesian total evidence majority-rule consensus tree, showing all compatible clades.

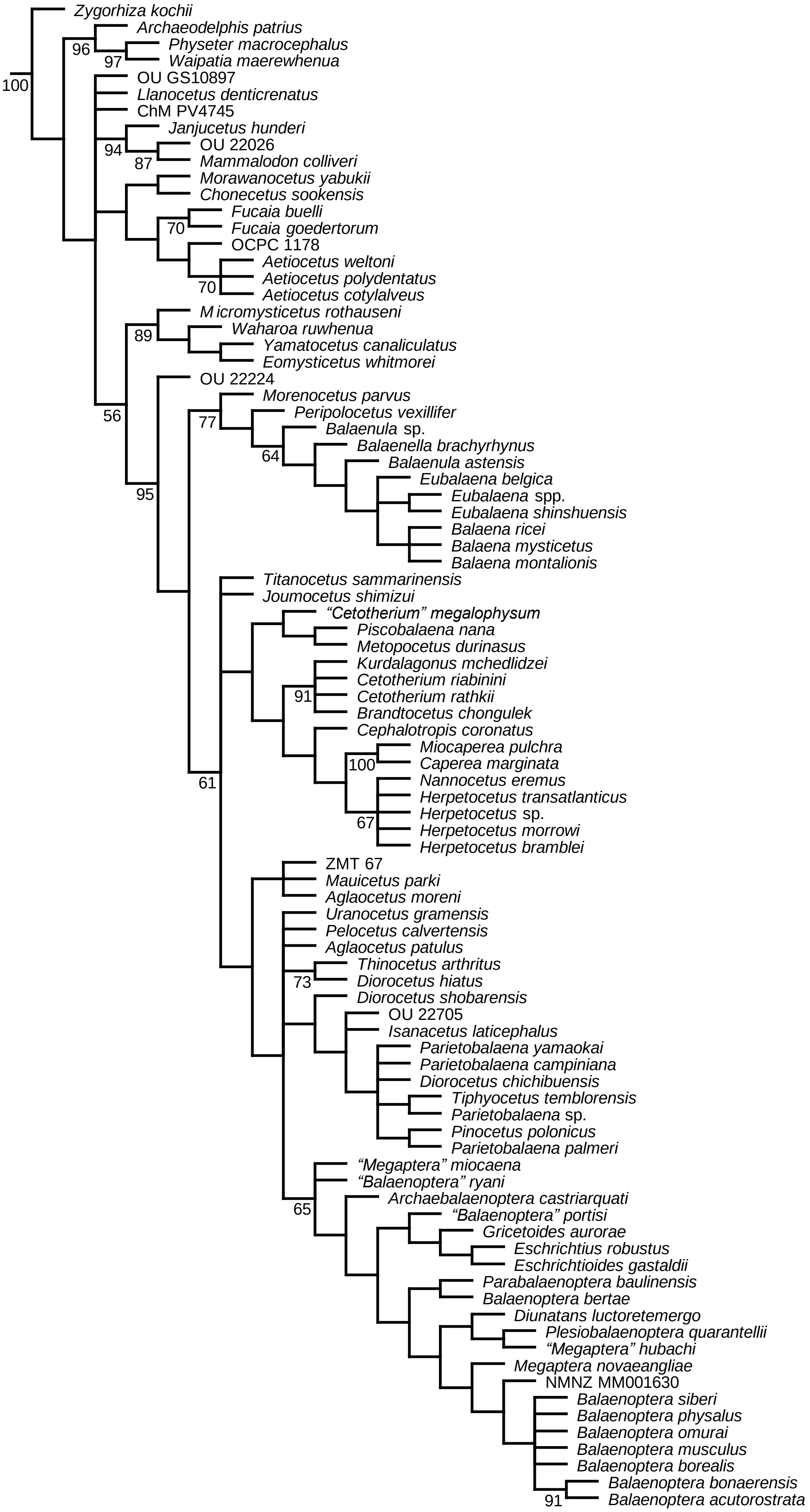

Supplementary Figure S2. Strict consensus of the equally weighted maximum parsimony analysis.

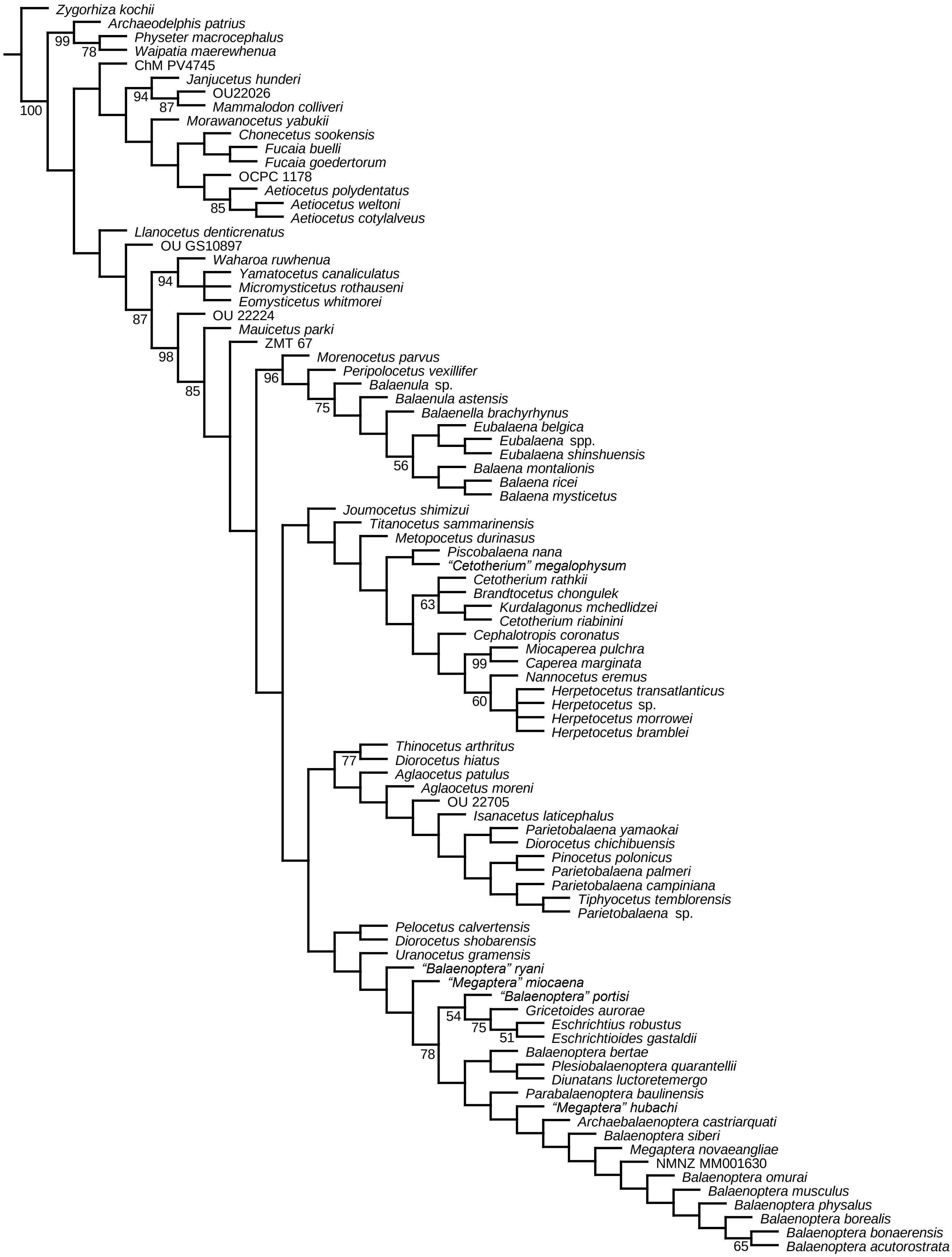

Supplementary Figure S3. Strict consensus of the maximum parsimony analysis under implied weighting.
